# NAT10-dependent *N*^4^‐acetylcytidine modification mediates PAN RNA stability, KSHV reactivation, and IFI16-related inflammasome activation

**DOI:** 10.1038/s41467-023-42135-3

**Published:** 2023-10-10

**Authors:** Qin Yan, Jing Zhou, Ziyu Wang, Xiangya Ding, Xinyue Ma, Wan Li, Xuemei Jia, Shou-Jiang Gao, Chun Lu

**Affiliations:** 1 Department of Gynecology, Women’s Hospital of Nanjing Medical University, Nanjing Maternity and Child Health Care Hospital, Nanjing Medical University, 210004 Nanjing, P. R. China; 2 Department of Microbiology, Nanjing Medical University, 211166 Nanjing, P. R. China; 3 Changzhou Medical Center, Nanjing Medical University, 211166 Nanjing, P. R. China; 4 Key Laboratory of Pathogen Biology of Jiangsu Province, Nanjing Medical University, 211166 Nanjing, P. R. China; 5 Tumor Virology Program, UPMC Hillman Cancer Center, and Department of Microbiology and Molecular Genetics, University of Pittsburgh, Pittsburgh, PA 15232 USA

**Keywords:** Virus-host interactions, Herpes virus

## Abstract

*N*-acetyltransferase 10 (NAT10) is an *N*^4^‐acetylcytidine (ac^4^C) writer that catalyzes RNA acetylation at cytidine N^4^ position on tRNAs, rRNAs and mRNAs. Recently, NAT10 and the associated ac^4^C have been reported to increase the stability of HIV-1 transcripts. Here, we show that NAT10 catalyzes ac^4^C addition to the polyadenylated nuclear RNA (PAN), a long non-coding RNA encoded by the oncogenic DNA virus Kaposi’s sarcoma-associated herpesvirus (KSHV), triggering viral lytic reactivation from latency. Mutagenesis of ac^4^C sites in PAN RNA in the context of KSHV infection abolishes PAN ac^4^C modifications, downregulates the expression of viral lytic genes and reduces virion production. NAT10 knockdown or mutagenesis erases ac^4^C modifications of PAN RNA and increases its instability, and prevents KSHV reactivation. Furthermore, PAN ac^4^C modification promotes NAT10 recruitment of IFN-γ-inducible protein-16 (IFI16) mRNA, resulting in its ac^4^C acetylation, mRNA stability and translation, and eventual inflammasome activation. These results reveal a novel mechanism of viral and host ac^4^C modifications and the associated complexes as a critical switch of KSHV replication and antiviral immunity.

## Abstract (web summary)

*N*-acetyltransferase 10 (NAT10) is an *N*^4^‐acetylcytidine (ac^4^C) writer, which catalyzes RNA acetylation at cytidine N^4^ position on RNAs. Here, the authors show that NAT10 catalyzes ac^4^C addition to a long non-coding RNA encoded by the oncogenic DNA virus Kaposi’s sarcoma-associated herpesvirus (KSHV), triggering viral lytic reactivation from latency, which promotes NAT10 recruitment of IFI16 mRNA, resulting in inflammasome activation.

## Introduction

The studies of RNA modifications in the virology field have blossomed in recent years. Among more than 150 RNA modifications with distinct properties and functions, *N*^6^-methyladenosine (m^6^A) is the most abundant and has been the most extensively explored^1^. *N*^4^-acetylcytidine (ac^4^C) has been identified as a novel mRNA modification catalyzed by *N*-acetyltransferase 10 (NAT10)^2,3^. ac^4^C, which refers to a modification occurring at the fourth position of cytidine (C) bases in RNA, influences the stability and translational efficiency of mRNAs and hence has been implicated in development and diseases^4^. Importantly, NAT10 is the only known human enzyme with both acetyltransferase and RNA binding activities^2^. In 2020, the first report on the role of ac^4^C in viral infection revealed that multiple cytidines on HIV-1 RNAs are acetylated to ac^4^C by cellular NAT10, while silent mutagenesis of these ac^4^C sites led to inhibited HIV-1 replication by reducing viral RNA stability^5^. Recently, ac^4^C on RNAs of enterovirus 71 and influenza A virus were identified^6,7^. However, whether ac^4^C modification occurs during infection of DNA viruses such as Kaposi’s sarcoma-associated herpesvirus (KSHV) remains unknown.

KSHV is causally associated with several malignancies, including Kaposi’s sarcoma (KS), primary effusion lymphoma (PEL), and multicentric Castleman’s disease (MCD)^8,9^. Like other herpesviruses, KSHV displays two distinct phases in the life cycle, including a latent phase and a productive lytic replication phase^10^. In most infected individuals, KSHV is predominantly latent, while the viral genome persists as a circular episome within the nucleus, with the expression of only a limited number of viral genes, including latency-associated nuclear antigen (LANA, encoded by ORF73), viral cyclin (vCyclin, encoded by ORF72), viral FLICE inhibitory protein (vFLIP, encoded by ORF71), kaposin (K12), and viral pre-microRNAs (pre-miRNAs)^10–12^. Upon intracellular or extracellular stimulation, KSHV latently infected cells can be reactivated into lytic replication, leading to the transcriptional activation in a cascade manner of three classes of lytic genes, characterized as immediate early (IE), early (E), and late (L) genes, and the production of viral progeny^12,13^. Both latent and lytic replication phases are crucial for the long-term persistence of KSHV in the host^14^. Therefore, understanding the mechanisms regulating the expression of viral genes in latency and lytic reactivation can provide insights into the KSHV life cycle and KSHV-associated pathogenesis.

Recent studies have revealed that during viral latency, KSHV alters m^6^A on transcripts of genes implicated in cellular transformation and viral latency^15^. We therefore hypothesized that the ac^4^C machinery might also modulate the expression of viral genes and KSHV lytic reactivation. Here, we found that NAT10-dependent ac^4^C modifications participated in the regulation of expression of viral genes and KSHV lytic replication and that this was involved with the RNA helicase domain and acetyltransferase domain of NAT10. Genome-wide analysis of KSHV transcripts identified NAT10-specific ac^4^C sites by acRIP-seq and NAT10-binding sites by RIP-seq. In particular, the PAN RNA, a KSHV-encoded long non-coding RNA (lncRNA), accumulated ac^4^C modifications at an exceedingly high level during viral reactivation. Genetic analysis of PAN ac^4^C sites showed that these modifications were critical for PAN RNA stability, the expression of viral late genes and virus production.

Upon sensing of viral DNA in the nucleus, IFN-γ-inducible protein-16 (IFI16) translocates to the cytoplasm to activate the NF-κB pathway and induce the transcription of type I interferon (IFN)^16,17^. IFI16 was reported to interact with the KSHV genome, triggering the formation of a functional inflammasome, i.e., caspase-1 activation and IL-1β cytokine maturation^18,19^. The KSHV genome was epigenetically silenced by IFI16-mediated H3K9me2/me3 deposition during de novo infection and latency^20^. Here we also observed that IFI16 mRNA was acetylated following KSHV reactivation. This process relied on NAT10-dependent PAN ac^4^C modification. Acetylated PAN assisted NAT10 to better recognize IFI16 mRNA, resulting in enhanced IFI16 translation efficiency and subsequent inflammasome activation.

Therefore, in this work, we reveal a novel mechanism by which a DNA virus hijacks a cellular protein to facilitate virus replication but, at the same time, triggers host innate immunity. We demonstrate that the acetyltransferase NAT10 catalyzes ac^4^C deposition on KSHV PAN RNA to promote its stability and KSHV reactivation, which assists NAT10 recruitment of IFI16 mRNA for innate inflammasome induction.

## Results

### NAT10 is required for KSHV lytic replication

**Fig. 1:**
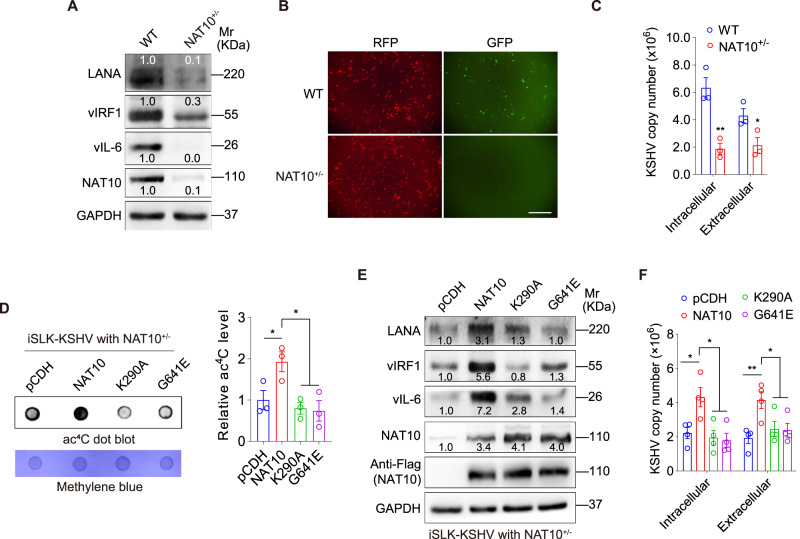
NAT10 promotes KSHV lytic replication. **A** iSLK-KSHV NAT10^+/−^ cells (**NAT10**^**+/**−^) and iSLK-KSHV NAT10^+/+^ cells (**WT**) as control were stimulated by doxycycline for 72 h and then examined for the expression of KSHV LANA, vIRF1, vIL-6, and cellular NAT10 by Western blot. **B** The red fluorescent protein (**RFP**) and green fluorescent protein (**GFP**) in cells shown in (**A**) were detected after doxycycline induction for 12 h. The scale bar is 40 μM. **C** By the assessment of ORF26, real-time DNA-PCR for cells shown in (**A**) was performed to detect intracellular and extracellular viral copy number after doxycycline stimulation for 72 h. *, *P* < 0.05 and **, *P* < 0.01 by two-sided *t*-test versus the WT group. **D** iSLK-KSHV cells with NAT10 KD (NAT10^+/−^) were transduced by the wild-type NAT10 (**NAT10**) or its mutant forms (**K290A** and **G641E**) or its control (**pCDH**). The total RNAs from indicated cells were subjected to an anti-ac^4^C-based dot blot assay to examine the RNA acetyltransferase activity of wild-type NAT10 and mutant NAT10. Methylene blue (MB) staining was used as the internal control (Left). Quantification of relative ac^4^C modification abundance was calculated by quantifying the gray value of ac^4^C/MB. The dots in the pCDH group were considered to be “1” for comparison. Plots indicate the number of replicates (Right). *, *P* < 0.05 by one-way ANOVA. **E** Cells shown in (**D**) were used to examine the expression of KSHV LANA, vIRF1, vIL-6, and cellular NAT10 or its Flag-tag by Western blot. **F** Cells shown in (**D**) were employed to detect intracellular and extracellular viral copy number by the assessment of ORF26 (real-time DNA-PCR) after doxycycline stimulation for 72 h. *, *P* < 0.05 and **, *P* < 0.01 by one-way ANOVA. Data represent mean ± SEM from *n* = 3 (**C**, **D**) or *n* = 4 (**F**) biological replicates shown as points. Source data are provided in a Source data file.

To investigate whether NAT10-dependent ac^4^C modification regulates the expression of KSHV genes and viral lytic replication, we employed the iSLK-KSHV system^21^. Treatment with doxycycline and sodium butyrate can trigger KSHV reactivation in iSLK-KSHV cells^21^. We utilized the CRISPR-Cas9 strategy to generate a heterozygous NAT10 cell line (NAT10^+/−^) (Supplementary Fig. 1A), resulting in significantly lower NAT10 expression in iSLK-KSHV cells (Fig. 1A)^4^. No difference was observed in cell proliferation, cell cycle and apoptosis between NAT10^+/+^ and NAT10^+/−^ cells (Supplementary Fig. 1B–D). Importantly, NAT10^+/−^ cells had dramatic reduction in the levels of KSHV gene expression including LANA, vIRF1 and vIL-6 (Fig. 1A and Supplementary Fig. 1E). In iSLK-KSHV cells, RFP serves as a marker for presence of viral genome of cells, while the expression of the enhanced green fluorescent protein (EGFP) cassette is under the control of polyadenylated nuclear RNA (PAN) promoter, which closely tracks KSHV lytic replication^21^. The number of EGFP-positive cells was markedly reduced in NAT10^+/−^ iSLK-KSHV cells compared to NAT10^+/+^ iSLK-KSHV cells (Fig. 1B), indicating that NAT10 is important for viral reactivation. In agreement with these results, both intracellular and extracellular KSHV genome copy numbers were decreased in NAT10^+/−^ cells (Fig. 1C).

NAT10 functions as an RNA acetyltransferase mainly through its RNA helicase domain and/or the acetyltransferase domain^22,23^. We overexpressed wild-type (WT) NAT10 or NAT10 mutants with mutations in K290A or G641E in NAT10^+/−^ iSLK-KSHV cells (Supplementary Fig. 1F) and examined viral reactivation after doxycycline and sodium butyrate treatment. The RNA acetyltransferase activity of WT NAT10 and its mutants were validated by anti-ac^4^C based dot blot in total RNA from iSLK-KSHV NAT10^+/−^ cells (Fig. 1D). We detected increased expression levels of viral proteins (Fig. 1E) as well as intracellular and extracellular KSHV genome copy numbers (Fig. 1F) in iSLK-KSHV NAT10^+/−^ cells with overexpression of WT NAT10 but not the K290A or G641E mutant. These data indicated NAT10 enhanced KSHV lytic replication and virion production.

### NAT10 catalyzes ac^4^C modifications on KSHV transcripts

**Fig. 2:**
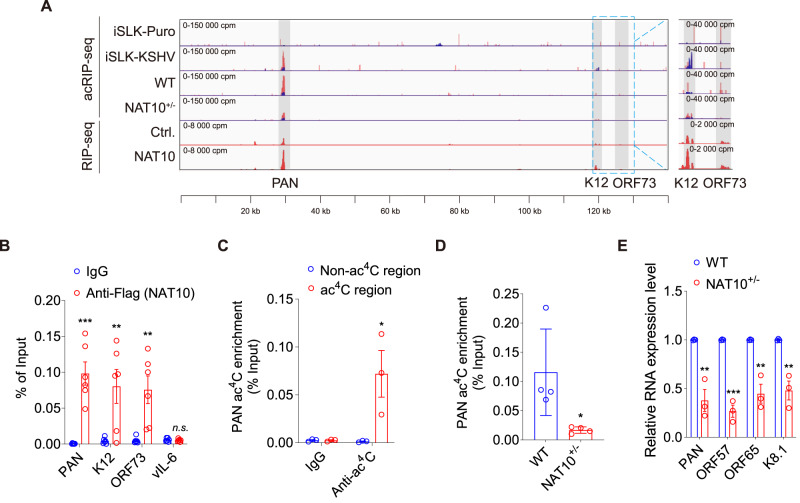
NAT10 catalyzes ac^4^C modification in KSHV transcripts. **A** ac^4^C peaks were mapped by acRIP-seq (**acRIP-seq**, upper lane) on iSLK-puro (**iSLK-Puro**) and iSLK-KSHV (**iSLK-KSHV**) cells, as well as iSLK-KSHV cells with (**NAT10**^**+/**−^) or without (**WT**) NAT10 knockdown. Abundant reads were mapped by RIP-seq (**RIP-seq**, lower lane) on iSLK-KSHV cells with the overexpression of NAT10-Flag (**NAT10**) or its control RFP-Flag (**Ctrl**.). Above cells were induced by doxycycline for 72 h, and sequencing reads were aligned with the KSHV genome. The ac^4^C sites were marked in gray, and sequencing read stacks were displayed in counts per million (cpm). Input and acRIP reads are marked as blue and red, respectively. **B** The iSLK-KSHV cells transduced by Flag-tagged NAT10 with doxycycline induction for 72 h were subjected to the **Anti-Flag (NAT10)** or immunoglobulin G (**IgG**) RNA immunoprecipitation, and the precipitated RNAs (**PAN,** **K12,** **ORF73** and **vIL-6**) were examined by RT-qPCR. **, *P* < 0.01 and ***, *P* < 0.001 by two-sided *t*-test versus the IgG group. *n.s*, not significant. **C** For iSLK-KSHV cells shown in (**B**), RNA immunoprecipitation was performed with anti-ac^4^C antibody (**Anti-ac**^**4**^**C**) or IgG (**IgG**), and subsequent RT-qPCR was used to detect ac^4^C enrichment on PAN RNA acetylated (**ac**^**4**^**C region**) or non-acetylated regions (**Non-ac**^**4**^**C region**). *, *P* < 0.05 by two-sided *t-*test versus the non-ac^4^C region. **D** acRIP-qPCR was performed in iSLK-KSHV cells with (**NAT10**^**+/**−^) or without (**WT**) NAT10 knockdown and doxycycline induction for 72 h to examine ac^4^C enrichment on PAN RNA. *, *P* < 0.05 by two-sided *t-*test versus the WT group. **E** RT-qPCR was performed to detect the mRNA expression levels of PAN, ORF57, ORF65 and K8.1 in iSLK-KSHV cells shown in (**D**). **, *P* < 0.01 and ***, *P* < 0.001 by two-sided *t-*test versus the WT group. Data represent mean ± SEM from *n* = 3 (**C**, **E**), *n* = 4 (**D**) or *n* = 6 (**B**) biological replicates shown as points. Source data are provided in a Source data file.

To map ac^4^C modification sites on KSHV transcripts, acRIP-seq was performed with a commercial ac^4^C antibody. 18S and 28S rRNA were used as positive/negative controls for acRIP-seq experiments, respectively (Supplementary Fig. 2A)^4,24^. We detected several ac^4^C peaks on KSHV PAN, ORF-K12 and ORF73 transcripts but not other viral transcripts, including vIL-6 (Fig. 2A and Supplementary Fig. 2A). PAN RNA had the most abundant peaks. Knockdown of NAT10 reduced the intensities of these peaks on viral transcripts (Fig. 2A). Similar to previous report^4^, the ac^4^C peaks on cellular mRNAs of iSLK cells were mainly localized to mRNA coding sequences (CDS) and more evenly on lncRNAs based on acRIP-seq results (Supplementary Fig. 2B).

Since the acetyltransferase activity of NAT10 relies on its RNA binding capacity, we performed RNA immunoprecipitation sequencing (RIP-seq) in iSLK-KSHV cells transduced with Flag-tagged WT NAT10 or a control Flag-tagged red fluorescent protein (RFP) after doxycycline and sodium butyrate induction to identify NAT10-binding viral transcripts. In agreement with the mapped ac^4^C sites, NAT10 bound to PAN, ORF-K12 and ORF73 transcripts with the highest binding peak detected on PAN RNA (Fig. 2A). As expected, the distribution of NAT10-binding sites from NAT10-RIP-seq on cellular mRNAs and lncRNAs were similar to the results of acRIP-seq (Supplementary Fig. 2B). We used Homer to identify the conserved sequence motifs of NAT10-dependent ac^4^C peaks and NAT10-binding sites on KSHV transcripts, and found that both ac^4^C sites and NAT10-binding sites had a C-rich sequence at both ends separated by two non-obligate nucleotides (CXXC, Supplementary Fig. 2C).

We further examined transcripts with differential acRIP-seq peaks and differential expression levels between NAT10^+/+^ iSLK-KSHV and NAT10^+/−^ iSLK-KSHV cells (Supplementary Fig. 3A, B). Gene ontology (GO) enrichment analysis revealed that genes in the “Defense response to virus” pathway were most enriched (Supplementary Fig. 3C), while NAT10-dependent genes were most enriched in pathways related to “Extracellular matrix organization” (Supplementary Fig. 3D). KEGG pathway analysis revealed enriched pathways implicated in virus infection (Supplementary Fig. 3E), while Hippo signaling pathway was the most enriched one in NAT10 knockdown cells (Supplementary Fig. 3F).

We performed RNA immunoprecipitation (RIP) followed by quantitative reverse transcription PCR (RT-qPCR) and successfully detected PAN, ORF-K12 and ORF73 but not non-acetylated viral transcript vIL-6 in NAT10 overexpressed (OE) iSLK-KSHV cells (Fig. 2B). Meanwhile, ac^4^C-RIP-qPCR confirmed that PAN RNA existed ac^4^C modification (Fig. 2C) while PAN RNA had reduced ac^4^C level in NAT10^+/−^ iSLK-KSHV cells (Fig. 2D). Furthermore, NAT10^+/−^ iSLK-KSHV cells also had reduced the expression levels of PAN RNA and KSHV lytic genes ORF57, ORF65 and ORF K8.1 (Fig. 2E).

### The ac^4^C modification promotes PAN RNA stability

**Fig. 3:**
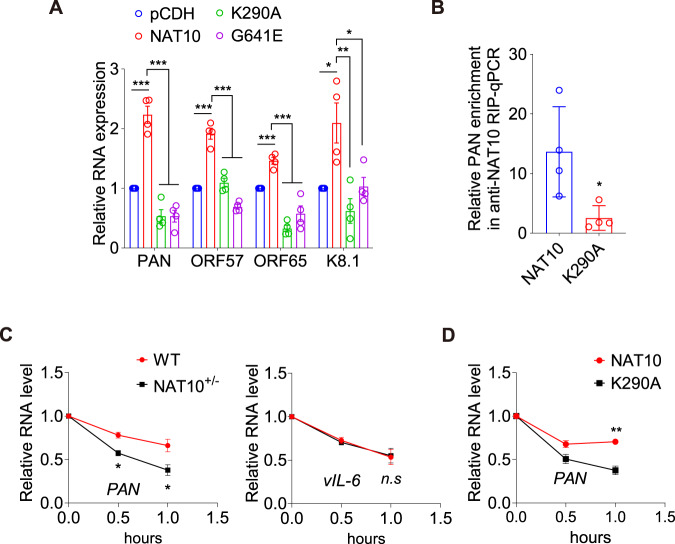
NAT10-dependent ac^4^C modification promotes PAN RNA stability. **A** RT-qPCR was performed to detect the mRNA expression levels of PAN, ORF57, ORF65 and ORF-K8.1 in iSLK-KSHV cells with the wild type (**NAT10**) or the mutant forms (**K290A,** **G641E**) of NAT10 overexpression, as well as their control (**pCDH**). *, *P* < 0.05, **, *P* < 0.01, and ***, *P* < 0.001 by one-way ANOVA versus the pCDH and NAT10 groups, respectively. **B** RIP assay was performed with anti-Flag antibody, and subsequent RT-qPCR was used to detect PAN RNA enrichment in iSLK-KSHV cells with the wild type (**NAT10**) or the mutant form (**K290A**) of NAT10 overexpression and doxycycline induction for 72 h. *, *P* < 0.05 by two-sided *t*-test versus the NAT10 group. **C** RNA decay assays with actinomycin D treatment for 0, 0.5, and 1 h were performed to detect the degradation rate of PAN RNA (Left) and vIL-6 mRNA (Right) in iSLK-KSHV NAT10^+/+^ (**WT**) or NAT10^+/−^ (**NAT10**^**+/**−^) cells. *, *P* < 0.05 by two-way ANOVA versus the WT group. *n.s*, not significant. **D** RNA decay assays with actinomycin D treatment for 0, 0.5, and 1 h were performed to detect the degradation rate of PAN RNA in iSLK-KSHV NAT10^+/−^ cells after rescuing WT (**NAT10**) or mutant (**K290A**) NAT10. **, *P* < 0.01 by two-way ANOVA versus the mutant K290A group. Data represent mean ± SEM from *n* = 3 (**C**, **D**) or *n* = 4 (**A**, **B**) biological replicates shown as points. Source data are provided in a Source data file.

Our results so far not only profiled NAT10-dependent ac^4^C modifications in viral and cellular transcripts but also revealed the regulation of PAN RNA by NAT10. To define the mechanism regulating PAN RNA expression by NAT10 and the ac^4^C modification, we examined the PAN RNA and the expression of other viral genes in iSLK-KSHV cells overexpressing WT NAT10 or its mutant of the RNA helicase domain or the acetyltransferase domain. WT but not the mutants significantly increased PAN expression as well as other viral genes (Fig. 3A). The RNA helicase domain mutant had reduced binding to the PAN RNA compared to the WT NAT10 (Fig. 3B). Since ac^4^C modification was recently reported to enhance the stability of RNAs, we examined RNA decay by treating the cells with actinomycin D. Compared to NAT10^+/+^ cells, NAT10^+/−^ cells stability PAN RNA but not that of the non-acetylated viral transcript vIL-6 was reduced (Fig. 3C). WT but not mutant NAT10 rescued the effect of NAT10^+/−^ cells (Fig. 3D). Collectively, these results revealed that NAT10 promoted PAN RNA stability and expression by increasing its ac^4^C modification.

### Loss of ac^4^C in PAN RNA inhibits KSHV lytic replication

**Fig. 4:**
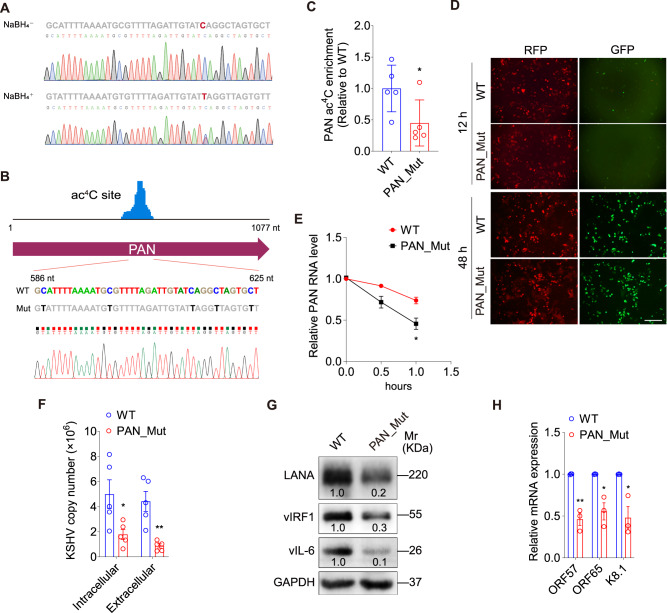
Mutagenesis of ac^4^C sites in PAN RNA diminishes KSHV lytic reactivation. **A** A C-T mismatch (marked as red) at C614 of PAN RNA was detected in doxycycline-induced iSLK-KSHV cells with (**NaBH**_**4**_^**+**^) or without (**NaBH**_**4**_^−^) NaBH_4_ treatment, which was verified by Sanger sequencing (below). **B** ac^4^C sites in PAN RNA (586 to 625 nt) were mutated from C (marked as blue) to T (marked as black) based on the KSHV BAC16 genome, which was verified by Sanger sequencing (below). **C** iSLK-KSHV cells with PAN WT (**WT**) or PAN point mutation (**PAN_Mut**) in the KSHV genome were induced by doxycycline for 72 h. Then acRIP-qPCR was performed to detect the ac^4^C enrichment on PAN RNA. *, *P* < 0.05 by two-sided *t-*test versus the WT group. **D** After doxycycline induction for 12 and 48 h in iSLK-KSHV cells with PAN WT (**WT**) or PAN point mutation (**PAN_Mut**), the RFP (marker for KSHV infection) and GFP (marker for KSHV lytic replication) expressions were observed under a fluorescence microscope. Scar bars, 40 μm. **E** RNA decay assays with actinomycin D treatment for 0, 0.5, and 1 h were performed to examine the degradation rate of PAN RNA in iSLK-KSHV cells with PAN WT (**WT**) or PAN point mutation (**PAN_Mut**). *, *P* < 0.05 by two-way ANOVA versus the WT group. **F** By the assessment of ORF26, real-time DNA-PCR for cells shown in (**C**) was performed to detect intracellular and extracellular viral copy number after doxycycline stimulation for 72 h. *, *P* < 0.05 and **, *P* < 0.01 by two-sided *t-*test versus the WT group. **G** The expression of LANA, vIRF1 and vIL-6 proteins in cells shown in (**C**) were detected by Western blot. **H** The mRNA expression levels of ORF57, ORF65 and K8.1 were examined by RT-qPCR in cells shown in (**C**). *, *P* < 0.05 and **, *P* < 0.01 by two-sided *t-*test versus the WT group. Data represent mean ± SEM from *n* = 3 (**E**, **H**) or *n* = 5 (**C**, **F**) biological replicates shown as points. Source data are provided in a Source data file.

Following identification of several ac^4^C peaks on KSHV PAN by acRIP-seq, we orthogonally confirmed one of ac^4^C sites by sodium borohydride (NaBH_4_) reduction of ac^4^C followed by sequencing (Fig. 4A). Multiple C to T mutations at the detected ac^4^C peak in the PAN RNA region were further introduced using the BAC16 KSHV reverse genetic system (Fig. 4B) and obtained the PAN_Mut iSLK-KSHV cells to directly examine the role of ac^4^C modification in PAN RNA in KSHV lytic replication. Introduction of C to T mutations reduced the ac^4^C modification in the PAN RNA (Fig. 4C) and the number of EGFP-positive cells (Fig. 4D). Also, the RNA stability of PAN was decreased after C-T mutations (Fig. 4E). Consistent with these results, these mutations reduced both intracellular and extracellular viral genome copy numbers (Fig. 4F), and the expression levels of viral proteins and transcripts (Fig. 4G, H). It was possible that the introduced C-T mutations might have changed the PAN RNA structure. However, when introducing compensatory mutations to maintain base-paired residues (Supplementary Fig. 4A, B), both ac^4^C-mutant and compensated-mutant PAN RNA reduced the intracellular viral genome copy numbers to the same extent (Supplementary Fig. 4C). RIP-qPCR were performed to evaluate the differences in the interaction of ac^4^C-mutant and compensated-mutant PAN with KSHV ORF26^25^. We did not detect any enrichment of HA-tagged ORF26 binding to the wide type PAN RNA as well as the ac^4^C-mutant and compensated-mutant PAN (Supplementary Fig. 4D), suggesting that the change in PAN RNA structure did not alter any putative interactions with ORF26. These results demonstrated that the ac^4^C site within PAN RNA was likely critical for viral replication.

### PAN acetylation recruits NAT10 to IFN-γ-inducible protein-16 (IFI16) mRNA

**Fig. 5:**
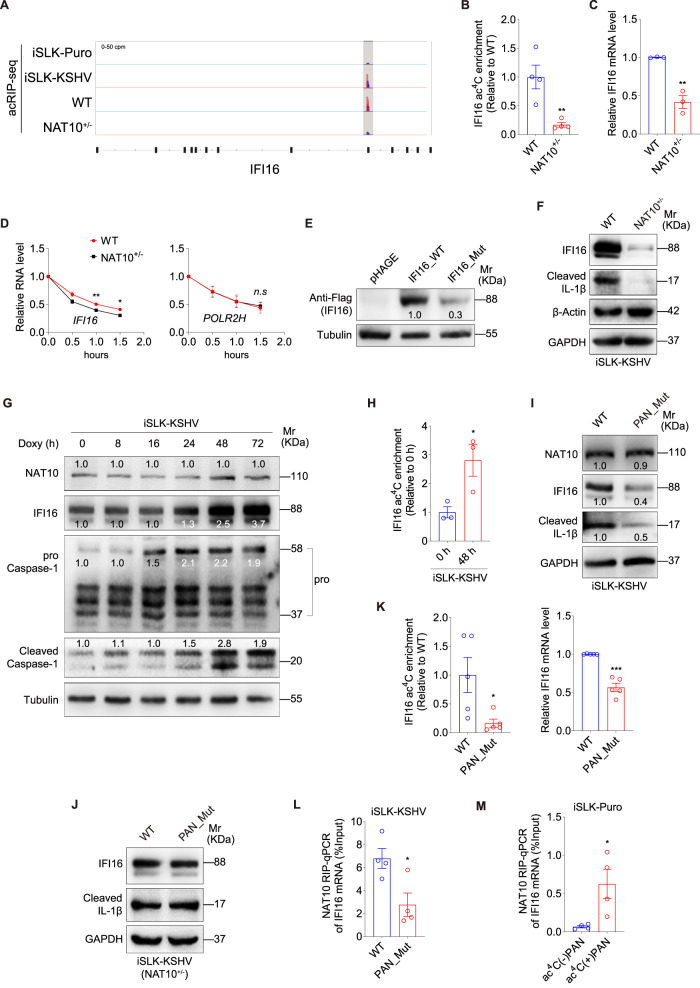
PAN recruits NAT10 to interact and acetylate IFI16 mRNA. **A** ac^4^C peaks of IFI16 mRNA mapped by acRIP-seq on cells shown in Fig. 2A, while sequencing reads were aligned with the human genome (hg19). Colors present the same as Fig. 2A. **B** acRIP-qPCR of IFI16 mRNA in iSLK-KSHV NAT10^+/+^ (**WT**) or NAT10^+/−^ (**NAT10**^**+/**−^) cells. **, *P* < 0.01 by two-sided *t-*test. **C** RT-qPCR for IFI16 mRNA in cells shown in (**B**). **, *P* < 0.01 by two-sided *t-*test. **D** RNA decay assays with actinomycin D treatment for 0, 0.5, 1.0 and 1.5 h for the degradation rate of *IFI16* mRNA in cells shown in (**B**). *POLR2H* mRNA served as the ac^4^C free negative control. *, *P* < 0.05 and **, *P* < 0.01 by two-way ANOVA versus the WT group. *n.s*, not significant. **E** Flag-tagged wide type (**IFI16_WT**), ac^4^C site-mutant (**IFI16_Mut**) IFI16 and the control (**pHAGE**) were transfected into HEK293T cells to detect IFI16 expression. **F** Western blot for IFI16 and cleaved IL-1β in cells shown in (**B**). **G** Western blot for NAT10, IFI16 and Caspase-1 during different KSHV reactivation time. **H** acRIP-qPCR of IFI16 mRNA during KSHV reactivation at 0 and 48 h. *, *P* < 0.05 by two-sided *t-*test. **I** Western blot for NAT10, IFI16 and cleaved IL-1β in iSLK-KSHV NAT10^+/+^ cells with PAN WT (**WT**) or PAN mutation (**PAN_Mut**). **J** Western blot for IFI16 and cleaved IL-1β in iSLK-KSHV NAT10^+/−^ cells with PAN WT (**WT**) or PAN point mutation (**PAN_Mut**). **K** acRIP-qPCR of IFI16 mRNA in cells shown in (**I**) (**Left**). IFI16 mRNA expression was analyzed from the input data (**Right**). *, *P* < 0.05 and ***, *P* < 0.001 by two-sided *t-*test. **L** Anti-NAT10 antibody-based RIP-qPCR was used to detect the interaction between IFI16 mRNA and NAT10 in cells shown in (**I**). *, *P* < 0.05 by two-sided *t-*test. **M** Anti-NAT10 antibody-based RIP-qPCR was used to detect the interaction between IFI16 mRNA and NAT10 in iSLK-Puro cells transfected with ac^4^C(−) or ac^4^C(+) PAN RNA. *, *P* < 0.05 by two-sided *t-*test. Data represent mean ± SEM from *n* = 3 (**C**, **D**, **H**), *n* = 4 (**B**, **L**, **M**) or *n* = 5 (**K**) biological replicates shown as points. Source data are provided in a Source data file.

A number of cellular genes has changed during KSHV reactivation or NAT10 knockdown (Supplementary Data 1). Host innate immune response is essential for controlling viral replication and infection. IFI16 functions as “defense response to virus”, “negative regulation of viral genome replication”, and “innate immune response” in GO analysis (Supplementary Data 1 and Supplementary Fig. 3). In addition, IFI16 mRNA could be detected in acRIP-seq in NAT10^+/+^ iSLK-KSHV cells but neither in NAT10^+/−^ iSLK-KSHV cells nor in KSHV-negative iSLK-puro cells (Fig. 5A). We hypothesized that NAT10 might assist IFI16 ac^4^C modification. Using ac^4^C-RIP-qPCR, we validated the acetylation and expression status of IFI16 mRNA. The results indicated that NAT10^+/−^ cells had decreased IFI16 mRNA acetylation and expression levels (Fig. 5B, C and Supplementary Fig. 5A). As it was reported that ac^4^C could affect mRNA stability and translation efficiency^4^, IFI16 mRNA but not the non-acetylated gene *POLR2H* indeed had increased degradation in NAT10^+/−^ cells (Fig. 5D)^4^. To examine the influence of ac^4^C on IFI16 translation efficiency, we overexpressed WT IFI16 and a mutant at the ac^4^C site without changing its amino acids sequence (Supplementary Fig. 5B) in an internal ribozyme entry site (IRES) based plasmid (Supplementary Fig. 5C) in 293T cells, which may lead to transiently ignoring the influence of RNA instability. While both WT and mutant IFI16 transcripts had similar GFP expression levels (Supplementary Fig. 5D), the mutant had a much lower protein expression level than that of the WT IFI16 (Fig. 5E).

IFI16 is a nuclear sensor, which activates the inflammasome in response to KSHV infection^18^. We found that NAT10^+/−^ cells had decreased cleaved-IL-1β expression level in addition to reduced IFI16 protein level (Fig. 5F). KSHV reactivation did not affect NAT10 expression but increased IFI16 expression and activated inflammasome after 48 h treatment of doxycycline (Fig. 5G). Consistent with these observations, the acetylation of IFI16 mRNA was increased following 48 h of doxycycline treatment (Fig. 5H).

To identify the relationship between KSHV PAN and host IFI16-inflammasome activation, we examined the IFI16 and inflammasome protein expression in iSLK PAN_Mut cells. We found that both IFI16 and inflammasome were inhibited following mutation of PAN ac^4^C site in KSHV genome in iSLK-KSHV NAT10^+/+^ cells (Fig. 5I) but not iSLK-KSHV NAT10^+/−^ cells (Fig. 5J). Both acetylated and total mRNA levels of IFI16 were decreased following PAN mutation (Fig. 5K). Importantly, the interaction between NAT10 and IFI16 mRNA was attenuated when the KSHV PAN ac^4^C site was mutated (Fig. 5L). Transfection of in vitro transcribed ac^4^C(+) PAN RNA into KSHV-negative iSLK-Puro cells enhanced the interaction between IFI16 mRNA and NAT10 (Fig. 5M). Taken together, these results showed that the acetylated PAN enhanced NAT10 recognition of the IFI16 mRNA, resulting in increased IFI16 protein level and innate inflammasome activation.

## Discussion

Emerging studies focused on RNA epitranscriptomics in recent years have revealed that the functions of mRNAs are regulated not only by the cognate sequences but also by a range of covalent modifications added to individual nucleotides^26,27^. As the most abundant internal mRNA modification found in mammalian cells and implicated in diverse cellular functions, m^6^A modifications in the genomes of RNA viruses and transcripts of a DNA virus play either a pro-viral or antiviral role^28–30^. In the context of KSHV, several groups have investigated the viral and cellular m^6^A epitranscriptomes during the KSHV life cycle and indicated that KSHV might hijack the m^6^A machinery to promote viral latency and induce cellular transformation depending on the cellular context^28,31^. However, whether other RNA modifications are also involved in the life cycle and pathogenesis of KSHV remains unknown.

Recently, ac^4^C has been described as the sole acetylation event in eukaryotic RNA at a level of ~1 ac^4^C residue per mRNA on cellular mRNAs^4^. Different from m^6^A modification, only one ac^4^C eraser (SIRT7) but no reader has been found so far^32^, while NAT10 has been identified as the only known acetyltransferase in human RNAs, which catalyzes ac^4^C deposition on different RNA substrates with acetyl-CoA serving as the acetyl donor^2,33^. In the case of cellular mRNAs, Arango et al. utilized transcriptome-wide approaches to investigate ac^4^C localization and function in mRNA and discovered that ac^4^C catalyzed by NAT10 within target mRNAs conferred enhanced stability and translation efficiency^4^. However, a recent study challenged the presence of ac^4^C in eukaryotic mRNA with a similar strategy involving reduction with sodium cyanoborohydride (NaCNBH_3_) followed by sequencing (termed ac^4^C-seq)^34^. To identify the reason for this inconsistency, Arango et al. employed sodium borohydride (NaBH_4_) reduction of ac^4^C followed by sequencing (termed RedaC:T-seq) and reanalyzed the ac^4^C-seq results. The authors found that sequencing depth was the major factor behind the inconsistency. Importantly, C:T mismatches previously detected by acRIP-seq were encompassed within the RedaC:T-seq peaks, thus supporting the accuracy and consistency of acRIP-seq and RedaC:T-seq^35^. We have also orthogonally validated the presence of one ac^4^C site in the PAN RNA region with NaBH_4_ treatment, which was consistent with the acRIP-seq result. However, other ac^4^C sites were not revealed, possibly because of the sequencing depth issue. A previous study identified ac^4^C residues in HIV-1 transcripts, and the loss of NAT10 expression resulted in a decline in HIV-1 gene expression through the stabilization of viral RNA transcripts^5^. Ac^4^C was subsequently characterized in the enterovirus 71 genome and found to promote viral replication^6^. Multiple ac^4^C sites were also detected within RNA regions of influenza A virus^7^. However, there is currently no report on the role of ac^4^C machinery in the transcriptome of any DNA viruses.

By genetic knockdown of NAT10 and mutagenesis of NAT10 residues required for RNA helicase or acetyltransferase function, we revealed that loss of ac^4^C modification in KSHV PAN RNA inhibited viral replication. Consistent with the findings in RNA viruses, depletion of the NAT10-dependent ac^4^C modification in the PAN RNA impeded viral RNA stability, resulting in reduced expression of numerous viral genes at both RNA and protein levels.

PAN RNA is a long non-coding polyadenylated transcript that facilitates viral lytic replication and suppresses the expression of host genes involved in the antiviral responses^36,37^. As an early lytic gene, PAN RNA is not expressed or is expressed at a low level during viral latency^38,39^. Following reactivation into the lytic phase, the PAN promoter is directly targeted by the immediate early transcription factor RTA^40^, resulting in an increased expression level of greater than 1000-fold, reaching as high as 1–5 × 10^5^ copies per cell^41^. In fact, as the most abundant viral transcript during lytic reactivation, PAN RNA is required for the expression of a subset of KSHV lytic genes, including K8.1, vIL-6, ORF6, ORF18 and ORF29, as well as the production of infectious virions^36,42^. Therefore, the dysregulation of  other KSHV lytic gene expression (Figs. 2E and 3A) is likely not directly caused in an ac^4^C-dependent way, but due to the subsequent effect of PAN RNA decline. In the current study, we showed that mutation of the ac^4^C site in PAN RNA resulted in reduced KSHV lytic replication and virion production. Although the mutation in the PAN RNA ac^4^C site (CT-mutation) may alter the structure of that region, it is unlikely to alter the interaction with ORF26^25^ and any unknown cellular proteins (Supplementary Fig. 4D). Hence, the expression and nuclear retention element (ENE), MTA responsive element (MRE) and other regions in PAN RNA that mediate its functions are unlikely to be affected.

Besides PAN RNA, our acRIP-seq data also showed several NAT10-dependent ac^4^C sites on ORF73 and K12 transcripts, whose ac^4^C peaks were less enriched than that of the PAN RNA. Different from PAN RNA, ORF73 and K12 are mainly expressed during KSHV latency. ORF73 is unchanged following lytic induction with 12-*O*-tetradecanoylphorbol-13-acetate (TPA)^43^ but is dramatically upregulated following tetracycline induction of RTA^44^. In contrast, ORF-K12 is significantly increased following TPA treatment or RTA induction^43,44^. Our findings implied that NAT10-mediated ac^4^C modifications on ORF73 and ORF-K12 genes may affect their expression levels; however, whether these modifications affect KSHV latent or lytic replication is still unknown. Given that the most abundant intracellular targets of NAT10 are 18S rRNA and tRNA^23,45,46^, KSHV reactivation might also be regulated by the abnormal tRNA and rRNA in the NAT10^+/−^ cells. The presence of ac^4^C on tRNAs or rRNAs might increase the fidelity during translation and improve translation efficiency^45,46^, which should be further explored in the future.

IFI16 and inflammasome activation were well studied in KSHV de novo infection^18,19^. Other than KSHV, IFI16 directly senses influenza A virus (IAV) RNA to enhance IFN-I production and inhibit IAV replication^47^. However, the mechanism by which IAV infection affects the IFI16 mRNA level remains unclear. Here we found that IFI16 mRNA was acetylated by NAT10 in the presence of KSHV PAN RNA. During KSHV reactivation, PAN RNA is highly expressed during KSHV early lytic replication and mediates the expression of other viral genes and viral production^36^. As a nuclear lncRNA, PAN RNA exerts its effects via interactions on chromatin^48^. Our results show that PAN RNA assists the interaction between IFI16 RNA and NAT10 during KSHV reactivation, leading to the ac^4^C modification of IFI16 RNA, which increases IFI16 mRNA and protein levels and inflammasome activation. As far as we know, this is the first description of such IFI16 mRNA modification by viral products and activation of inflammasome. It would be interesting to examine whether a similar mechanism is also present in other viral infections.

The box C/D snoRNA U13 is required for human 18S rRNA acetylation through base-pairing between U13 and the 3′ end of 18S rRNA^23^. We analyzed the potential base pairing between PAN RNA and IFI16 RNA but failed to identify such an interaction. We speculated that NAT10 might have a low binding affinity for IFI16 RNA. However, the interaction of IFI16 RNA with PAN RNA might have helped recruit NAT10 to the IFI16 RNA, while the underlying sensing mechanism remains unknown. Thus, while KSHV utilizes NAT10 to acetylate PAN RNA to promote replication, the interaction of the IFI16 RNA with the extremely abundant PAN RNA might lead to IFI16 RNA acetylation, stabilization and enhanced translation, resulting in inflammasome activation. Therefore, our results provide insights into an alternative mechanism underlying innate inflammasome activation during KSHV replication and possibly de novo infection.

KSHV de novo infection and lytic replication induce host inflammation^49^, but inflammation has been shown to promote viral lytic replication^50^, indicating that KSHV might hijack the inflammasome to promote lytic replication. Here we described an interplay scenario between KSHV replication and host response. Both pathogens and hosts evolve a mechanism to counter one another and reach a balance. It should be noted that *N*^*4*^-acetylcytidine, as a nucleoside metabolite itself, induces the activation of the inflammasome in microglia or THP-1 cells^51,52^. Therefore, the reduced inflammasome activation in NAT10^+/−^ cells might be a consequence of reduced ac^4^C release from cells, which should be further investigated.

**Fig. 6:**
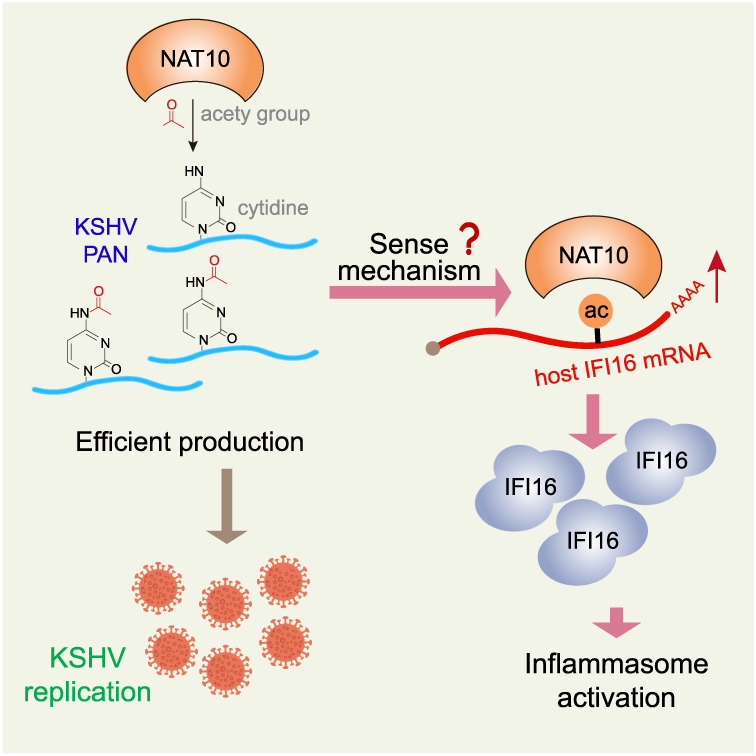
Schematic model for NAT10-dependent ac^4^C modification in KSHV reactivation and inflammasome induction. The acetyltransferase NAT10 catalyzes ac^4^C deposition on KSHV PAN RNA, with acetyl-CoA serving as the acetyl donor. Genetic depletion of NAT10 results in ac^4^C loss and instability of PAN, preventing the latency-lytic transition of KSHV and virion production. Acetylated PAN may assist NAT10 to better recognize IFI16 mRNA with an unknown sensing mechanism, resulting in increased IFI16 translation efficiency and innate inflammasome induction.

Taken together, we have identified multiple KSHV transcripts that are modified with ac^4^C by NAT10 and that the ac^4^C modification in PAN RNA is critical for KSHV lytic replication. Knockdown of NAT10 or mutation of the ac^4^C site in PAN RNA led to decreased RNA stability and reduced expression of viral lytic genes. Cellular IFI16 mRNA was modified with ac^4^C, which relied on NAT10-dependent PAN ac^4^C modification. PAN RNA assisted in NAT10 recruitment to IFI16 mRNA, resulting in IFI16 ac^4^C modification and increased expression of mRNA and protein, and activation of innate inflammasome (Fig. 6). Since KSHV lytic replication is important for KSHV spread and KS progression, our findings provide not only the evidence that there is an interplay among ac^4^C modifications and replication of DNA viruses and innate inflammasome activation but also identify a promising therapeutic target for KSHV-related malignancies.

## Methods

### Cell culture and reagents

iSLK-KSHV cells were cultured in high-glucose DMEM supplemented with 10% fetal bovine serum (FBS), 1 μg/mL puromycin (Beyotime, China), 250 μg/mL G418 (Biofroxx, China), and 1.2 mg/mL hygromycin B (BBI, China), while iSLK-Puro cells as the KSHV-negative control were maintained under the same conditions as iSLK-KSHV cells except for the absence of hygromycin B supplement^21^. iSLK-KSHV contains a cassette encoding RFP to monitor genome activation. iSLK-KSHV cells were selected with hygromycin to achieve stable latent infection. The above cell lines are kindly provided by Dr. Ke Lan from the State Key Laboratory of Virology, Wuhan University (Wuhan, China). HEK293T was purchased from ATCC (CRL-11268) and transfected with the Lipofectamine 2000 Reagent (Invitrogen, USA). All of the cell lines were negative for mycoplasma contamination.

### Plasmid construction

The plasmid with Flag-tag (pCDH-Flag-RFP), the NAT10 overexpression plasmid (pCDH-Flag-NAT10) and the NAT10 mutant plasmids (pCDH-Flag-NAT10 mut 1 and pCDH-Flag-NAT10 mut 2) were constructed based on the lentiviral expression vector (pCDH-CMV-MCS-EF1-RFP) (Sangong Biotech Co., Ltd., Shanghai). The sequence of NAT10 open reading frame (ORF) was referenced from pENTER-NAT10 (AH874058, Weizhen Biotechnology Co., Ltd., Shandong) with a series of Flag- and His-tag connected to the C-terminus of NAT10 ORF. The lysine at position 290 and the glycine at position 641 of the amino acids encoded by NAT10 were mutated to alanine (K290A) and glutamic acid (G641E), respectively. The ORF26 overexpression plasmid (pCDH-ORF26-HA) was constructed by Sangong Biotech Co., Ltd. All plasmids were prepared using the Vazyme FastPure Plasmid Mini Kit.

### Generation of NAT10-knockdown and overexpressing iSLK-KSHV cells

NAT10^+/−^ iSLK-KSHV cells were obtained by lentivirus transduction, which were produced by the lentiviral expression vector LentiCRISPR-NAT10 that encoded Cas9 and a single guide RNA (sgRNA): 5’-TGAGTTCATGGTCCGTAGG-3’. Two days after transduction, the cells were sorted with BD FACS ARIA II SORP and seeded into 96-well plates. iSLK-KSHV cells obtained by LentiCRISPRv2 transduction were used as a control. To verify the mutation efficacy, genomic DNA was extracted from a monoclonal cell line using a genomic DNA extraction kit. The genomic region flanking the Cas9 target site of each NAT10^+/−^ cell line was amplified by PCR and cloned into the Not I/BamH I site of pHAGE. Bacterial cell clones of pHAGE genomic region plasmids isolated from each CRISPR knockdown clone were subjected to Sanger sequencing. The lentiviral expression vector, pCDH-Flag-NAT10 or pCDH-Flag-RFP, was used to generate iSLK-KSHV cells expressing Flag-NAT10 or Flag-RFP as a control.

### acRIP-seq

acRIP-seq was mainly completed by Guangzhou Epibiotek Co., Ltd. Briefly, total RNA was separated and purified by the phenol-chloroform method, and rRNAs were removed by QIAseq FastSelect-rRNA HMR Kits (Qiagen, Cat. No: 334385); the concentration of total RNA was measured by Qubit RNA HS assay kit (Invitrogen, Q32852). Then, 150 μg of total RNA was taken for DNA digestion and fragmented with Epi^TM^ ac^4^C immunoprecipitation kit and then was purified and recovered by Zymo RNA clean and concentrator-25 kit. Next, RNA mixed with ac^4^C antibody and washed protein G-magnetic beads (Invitrogen™, Cat. No: 10004D) in precipitation buffer were incubated together at 4 °C for 4–6 h. After magnetic separation, the supernatant was removed and the magnetic beads were washed twice with precipitation buffer. The supernatant was discarded and RNA purification product, 5 × precipitation buffer and RNase inhibitor were added to incubate overnight at 4 °C. On the second day, magnetic beads were washed twice with low-salt precipitation buffer and high-salt buffer orderly. The ac^4^C-enriched RNA was eluted from the beads in the RLT buffer supplied in the RNeasy Mini Kit (QIAGEN, 74106). The RNA-seq library was prepared by NEBNext® Ultra^TM^ II RNA Library Prep Kit for Illumina® (NEB, E7775). PCR amplification was performed to enrich the library fragments, and the DNA purification magnetic beads were used to purify and screen the fragments. Beads purification library fragments were obtained by Bioptic Qsep100 Analyzer for quality inspection in the library. Both the input samples without IP and the ac^4^C IP samples were subjected to 150-bp, paired-end sequencing on an Illumina NovaSeq 6000 sequencer. The sequencing depth is 10G base.

### ac^4^C-RIP-qPCR

Total RNA was isolated from cells using TRIzol reagent (Invitrogen) and then fragmented into ~100 nucleotides using RNA fragmentation reagent (Invitrogen) and incubated at 95 °C. A total of 10 μg anti-ac^4^C antibody (ab252215, Abcam) or anti-rabbit IgG antibody was pre-incubated with 50 μL Protein A/G magnetic beads in IP buffer (150 mM NaCl, 0.1% NP-40, 10 mM Tris-HCl, pH 7.4) at room temperature for 0.5 h. Then, 300 μg of RNA fragments were added to the pre-incubated beads and incubated at 4 °C for 3 h. The bound ac^4^C-modified RNA was eluted for analysis by RT-qPCR as described below. Equal amounts of RNA fragments (not subjected to immunoprecipitation) were used as the input controls.

### NAT10 RIP-seq

NAT10 RIP-seq assay on iSLK-KSHV cells was performed by SeqHealth Co., Ltd. (Wuhan, China). The cells were treated with lysis buffer, and 10% cell lysis was used as an “input”, while 80% cell lysis was used in immunoprecipitation reactions with anti-NAT10 antibody and referred to as “IP”. The remaining 10% cell lysis was incubated with rabbit IgG (Cell Signaling Technology) as a negative control and named as “IgG”. The RNA from input and IP groups were extracted using TRIzol reagent (Invitrogen). The stranded RNA sequencing library was constructed by KC-Digital^TM^ Stranded mRNA Library Prep Kit for Illumina® (DR08502, SeqHealth Co., Ltd., Wuhan, China) according to the manufacturer’s instruction, which eliminated duplication bias in PCR and sequencing steps by a unique molecular identifier (UMI) of eight random bases to label the pre-amplified cDNA molecules. The library products corresponding to 200–500 bps were enriched, quantified and sequenced on a Novaseq 6000 sequencer (Illumina) with PE150 model.

### RIP-qPCR

RIP assay was performed using Magna RIP Kit (17-701, Millipore) according to the manufacturer’s instructions. Briefly, 5 μg anti-DDDDK (MBL, Japan), anti-HA (MBL, Japan) or anti-Mouse IgG (Millipore, Germany) incubated with 50 μL magnetic beads were added into cell lysates (~10^7^ cells per sample). Then, the RNA-protein IP complexes were washed 6 times and incubated with proteinase K to isolate immunoprecipitated protein-bound RNA. Finally, the released RNA was purified by phenol-chloroform RNA extraction for qPCR analysis.

### Illumina sequencing and bioinformatic data analysis

acRIP-seq data processing Cutadapt (v2.5) was used to trim adapters and filter for sequences. The remaining reads were aligned to the human Ensemble genome (GRCh38) by Hisat2 aligner (v2.1.0) with default parameters, which were removed for subsequent alignment to the KSHV genome (https://www.ncbi.nlm.nih.gov/nuccore/NC_009333.1) with the modified maximum mismatch penalties (=2). ac^4^C peaks were identified using exomePeak R package (v2.13.2) under parameters: PEAK_CUTOFF_PVALUE = 0.05, PEAK_CUTOFF_FDR = NA, FRAGMENT_LENGTH = 200, while the differential ac^4^C peak were indicated with parameters: PEAK_CUTOFF_PVALUE = 0.05, PEAK_CUTOFF_FDR = NA, FRAGMENT_LENGTH = 200. Gene ontology (GO) analysis and Kyoto encyclopedia of genes and genomes (KEGG) analysis were performed using clusterprofile R package (v3.6.0), and ac^4^C-RNA-related genomic features were visualized using Guitar R package (v1.16.0). ac^4^C peaks that with *P*-value < 0.05 were chosen for the de novo motif analysis using homer (v4.10.4) under parameters: -len 12 -rna. Raw sequencing data was initially filtered by Trimmomatic (version 0.36). Low-quality reads were discarded and the reads contaminated with adapter sequences were trimmed. Clean Reads were further treated with in-house scripts to eliminate duplication bias introduced in library preparation and sequencing. In brief, clean reads were firstly clustered according to the UMI sequences, in which reads with the same UMI sequence were grouped into the same cluster. Reads in the same cluster were compared to each other by pairwise alignment, and then reads with sequence identity over 95% were extracted to a new sub-cluster. After all sub-clusters were generated, multiple sequence alignment was performed to obtain one consensus sequence for each sub-cluster. At last, any errors and biases introduced by PCR amplification or sequencing were eliminated.

The de-duplicated consensus sequences were used for protein binding site analysis, which were mapped to the reference genome of KSHV using STAR software (version 2.5.3a) with default parameters. RSeQC (version 2.6) and exomePeak (version 3.8) were used for reads distribution analysis and peak calling, respectively. Peaks were annotated by bedtools (version 2.25.0), while the deepTools (version 2.4.1) was used for peak distribution analysis. Using the Fisher test, the differential binding peaks were identified by a Python script. Sequence motifs enriched in peak regions were identified using Homer (version 4.10). GO analysis and KEGG enrichment analysis for annotated genes were implemented by KOBAS software (version 2.1.1) with a corrected *P*-value cutoff of 0.05 to judge statistically significant enrichment. All deep sequencing data have been deposited at the NCBI GEO database under accession numbers GEO: GSE174161 and GSE173889.

### NaBH_4_ treatment

Total RNA was purified from iSLK-KSHV cells induced for 72 h using TRIzol reagent (Invitrogen). Then, 1 M NaBH_4_ (71320-25 G, Sigma) was added to 2 μg RNA in nuclease-free H_2_O to a final concentration of 100 mM. NaBH_4_-treated RNA samples were incubated at 37 °C for 1 h, and NaBH_4_ was then quenched with 1 M HCl (15 μL) and neutralized by the addition of 1 M Tris-HCl (pH 8.0) buffer (15 μL).

### KSHV reactivation in wild-type and NAT10^+/−^ iSLK-KSHV cells

iSLK-KSHV NAT10^+/−^ cells and the control cells were seeded in a 6-well plate at a density of 1 × 10^5^ cells/well, and 1 μg/mL doxycycline was further added to stimulate KSHV lytic reactivation for 72 h. Above induced cells and their supernatant were collected. Then, 1 μL DNase I was added to the supernatant and bathed in 37 °C water for 1 h to digest linear DNA molecules. Then, KSHV progeny virus DNA in the supernatant was extracted with a micro-sample genomic DNA extraction kit and determined by absolute quantification. To establish a standard curve of Ct value versus genome copy number, pcDNA3.1-ORF26 plasmid (KSHV minor capsid gene ORF26) was serially diluted to the range of 4 × 10^2^ to 4 × 10^7^ molecules/µL. Relative viral protein and mRNA levels were detected by Western blot and RT-qPCR, respectively.

### RNA decay assays

Cells were seeded in a 12-well plate at a density of 5 × 10^4^ cells/well. On the second day, when the cell confluence reached about 40%, 1 µg/mL doxycycline was added to the culture medium for 72 h. Then, 5 µg/mL Actinomycin D was added to the supernatant to inhibit mRNA transcription. Cells were harvested after Actinomycin D treatment for 0, 0.5, 1 and 1.5 h, and total cell RNA was extracted with TRIzol reagent. Viral RNA levels were assessed by RT-qPCR and analyzed using the 2^−ΔΔCt^ method. The residual RNAs were normalized to 0 h.

### RNA extraction and RT-qPCR analysis

Total RNA was extracted using TRIzol reagent (Invitrogen) and then reverse transcribed into cDNA using HiScript III RT SuperMix (Vazyme Biotech Co., Ltd.). RT-qPCR analysis was performed with ChamQ SYBR qPCR Master Mix (Vazyme Biotech Co., Ltd.) on StepOnePlus^TM^ Real-Time PCR System (Applied Biosystems). The results were analyzed using the 2^−ΔΔCt^ method and standardized with GAPDH. All specific RT-qPCR primers were listed in Supplementary Table 1.

### Western blot and antibodies

Cells were lysed with RIPA lysis buffer (1 × SDS, 1 × phosphatase inhibitor cocktail, 1 × protease inhibitors) to obtain protein samples. Then, the samples were separated with SDS-PAGE and transferred to the PVDF membrane. The blots were visualized by ECL after incubating with the primary antibodies and secondary antibodies. Anti-GAPDH (sc-47724; 1:500), anti-β-actin (sc-47778; 1:500) and anti-Tubulin (sc-23948; 1:500) were purchased from Santa Cruz Biotechnology, while anti-Flag was purchased from MBL (M185-3L, Tokyo, Japan; 1:1000). Antibodies against NAT10 (ab194297; 1:1000), LANA (ab4103; 1:1000), IFI16 (ab169788; 1:1000) and ac^4^C (ab252215; 1:1000) were from Abcam. Anti-pro Caspase-1+p10+p12 (ab179515; 1:1000) was also obtained from Abcam. IL-1β (3A6) mouse mAb (12242; 1:1000) and cleaved Caspase-1 (Asp296) (E2G2I) rabbit mAb (89332; 1:1000) were from Cell Signaling Technology. The monoclonal rabbit anti-vIL-6 antibody (1:500) was kindly provided by Dr. Robert Yarchoan from the Center for Cancer Research, National Cancer Institute (Bethesda, Maryland, USA)^53,54^, and polyclonal rabbit anti-vIRF1 antibody (1:300) was from Dr. Gary Hayward from Viral Oncology Program, The Johns Hopkins School of Medicine (Baltimore, Maryland, USA)^55–58^.

### Construction of PAN mutant cells

PAN mutant plasmid was constructed with a two-step Red mutagenesis system^59^. The kanamycin sequence carrying PAN homologous arms was amplified by PCR and transformed into GS1783 by electroporation to complete the first recombination. Then, under the induction of arabinose, the kanamycin sequence was cut off for the second recombination. PCR primers for mutagenesis are listed in Supplementary Table 1. After PAN_Mut BAC16 plasmid extraction and transfection, iSLK-KSHV cells with PAN mutant were obtained after the addition of hygromycin B for selection.

### In vitro transcript of ac^4^C-modified PAN RNA

T7 RNA polymerase promoter and PAN sequence were constructed into pHAGE plasmid. Then the plasmid was linearized with *Bam* HI. After purification, PAN was in vitro transcribed with CTP or ac^4^CTP (synthesized based on a published protocol^60^) and purified according to the manufacturer’s instructions (ThermoFisher, K0441). RNA integrity was assessed by 1% agarose after purification.

### Cell cycle analysis and apoptosis assay

The cell cycle was analyzed by 50 µg/mL propidium iodide (PI) staining. Apoptotic cells were detected by Annexin V-FITC Apoptosis Detection Kit (C1062, Beyotime, China) according to the manufacturer’s instructions. Flow cytometry was performed by BD FACSCalibur (BD Biosciences, San Jose, CA) and analyzed by ModFit LT 5.0 (Verity Software House) and FlowJo V10.8.0 (BD Biosciences) for cell cycle analysis and apoptosis assay, respectively, while the gating strategy was defined as compared to non-stained cells.

### Statistical analysis

GraphPad Prism 8 was used to calculate the standard deviation of the data. An unpaired two-tailed *t-*test was used for two groups comparisons. One- or two-way ANOVA was used for multiple comparison test as appropriate. *P* < 0.05 was considered statistically significant. All the experiments were repeated no less than three biological times. The data shown represent mean ± SEM from three independent experiments at least.

### Reporting summary

Further information on research design is available in the Nature Portfolio Reporting Summary linked to this article.

### Supplementary information

Supplementary Information

Reporting Summary

Description of Additional Supplementary Files

Supplementary Data 1

### Source data

Source Data

## Data Availability

The acRIP-seq and NAT10 RIP-seq data generated in this study have been deposited in the NCBI Gene Expression Omnibus (GEO) database under accession numbers GSE174161 and GSE173889, respectively. The acRIP-seq and NAT10 RIP-seq data based on KSHV genome reference (https://www.ncbi.nlm.nih.gov/datasets/genome/GCF_000838265.1/) and human genome reference (https://www.ncbi.nlm.nih.gov/datasets/genome/GCF_000001405.13/), respectively. All the other data associated with this study are shown in the manuscript, Supplementary Information, and Source Data file. Source data are provided with this paper.
