# Supplementary material for: NAT10-dependent N4‐acetylcytidine modification mediates PAN RNA stability, KSHV reactivation, and IFI16-related inflammasome activation
Source: Nat Commun. 2023 Oct 10;14:6327. doi: 10.1038/s41467-023-42135-3 (PMC10564894; doi:10.1038/s41467-023-42135-3)
Supplement: Supplementary file 1 — Supplementary Information [file 41467_2023_42135_MOESM1_ESM.pdf]

## Supplementary Information

# NAT10-dependent $N^4$-acetylcytidine modification mediates PAN RNA stability, KSHV reactivation, and IFI16-related inflammasome activation

Qin Yan, [1, 2, 3, 4 †] Jing Zhou, [2 †] Ziyu Wang, [2 †] Xiangya Ding, [1 †] Xinyue Ma, [2] Wan Li, [2, 3, 4] Xuemei Jia, [1 *] Shou-Jiang Gao, [5] and Chun Lu [1, 2, 3, 4 *]

[1] Department of Gynecology, Women's Hospital of Nanjing Medical University, Nanjing Maternity and Child Health Care Hospital, Nanjing Medical University, Nanjing 210004, P. R. China

[2] Department of Microbiology, Nanjing Medical University, Nanjing 211166, P. R. China

[3] Changzhou Medical Center, Nanjing Medical University, Nanjing 211166, P. R. China

[4] Key Laboratory of Pathogen Biology of Jiangsu Province, Nanjing Medical University, Nanjing 211166, P. R. China

[5] Tumor Virology Program, UPMC Hillman Cancer Center, and Department of Microbiology and Molecular Genetics, University of Pittsburgh, Pittsburgh, PA 15232, USA

† These authors contributed equally to this work

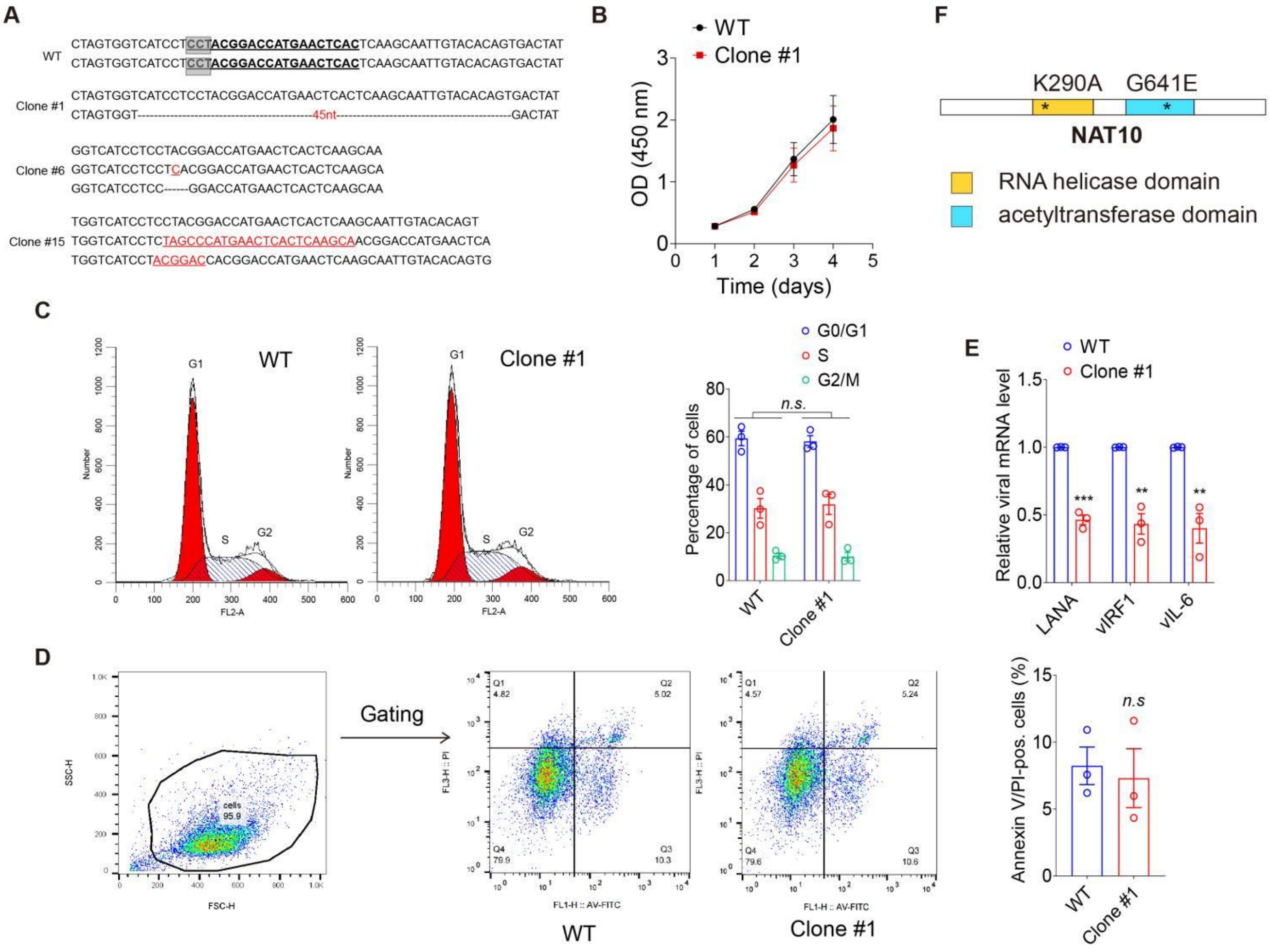
A
WT
CTAGTGGTCATCCTCCTACGGACCATGAACTCACTCAAGCAATTGTACACAGTGACTAT
CTAGTGGTCATCCTCCTACGGACCATGAACTCACTCAAGCAATTGTACACAGTGACTAT
Clone #1
CTAGTGGTCATCCTCCTACGGACCATGAACTCACTCAAGCAATTGTACACAGTGACTAT
CTAGTGGT--------45nt--------GACTAT
Clone #6
GGTCATCCTCCTACGGACCATGAACTCACTCAAGCAA
GGTCATCCTCCTCACGGACCATGAACTCACTCAAGCA
GGTCATCCTCC------GGACCATGAACTCACTCAAGCAA
Clone #15
TGGTCATCCTCCTACGGACCATGAACTCACTCAAGCAATTGTACACAGT
TGGTCATCCTCTAGCCCATGAACTCACTCAAGCAACGGACCATGAACTCA
TGGTCATCCTACGGACCACGGACCATGAACTCAAGCAATTGTACACAGTG
B
WT
Clone #1
OD (450 nm)
Time (days)
F
K290A
G641E
NAT10
RNA helicase domain
acetyltransferase domain
C
WT
Clone #1
G1
S
G2
Number
FL2-A
G0/G1
S
G2/M
n.s.
Percentage of cells
E
WT
Clone #1
Relative viral mRNA level
***
**
LANA
vIRF1
vIL-6
D
cells
95.9
SSC-H
FSC-H
Gating
Q1
4.82
Q2
5.02
Q3
10.3
Q4
79.9
Q1
4.57
Q2
5.24
Q3
10.6
Q4
79.6
FL3-H :: PI
FL1-H :: AV-FITC
WT
Clone #1
Annexin V/PI-pos. cells (%)
n.s

**Supplementary Figure 1. Generation of NAT10 knockdown cells based on CRISPR/Cas9 editing technology.**

**(A).** Diagram of the CRISPR-targeted genomic exon 5 region of NAT10 in iSLK-KSHV cells (**WT**) and three CRISPR-Cas9 clones. The mutated sequences of CRISPR Clones #1, #6, and #15 were shown. The Cas9 PAM (5'-NGG-3') was shown in grey boxes, while the indels and substitutions were shown in red. The sequences targeted by the gRNAs were marked underlined and bolded. Unless otherwise specified, the $NAT10^{+/-}$ clones used in this study was Clone #1.

**(B).** CCK8 assays were performed with cells shown in Clone #1 on days 1, 2, 3 and 4. Data were analyzed by two-way ANOVA versus the WT group.

**(C).** Cell cycle assays were performed with cells shown in Clone #1. *n.s*, not significant by two-sided *t* test.

**(D).** Cell apoptosis assays were performed with cells shown in Clone #1. Gating strategy is shown in the left lane. *n.s*, not significant by two-sided *t* test.

**(E).** Viral transcripts were quantized by RT-qPCR in $NAT10^{+/+}$ (**WT**) or $NAT10^{+/-}$ (**Clone #1**) cells. **, $P < 0.01$ and ***, $P < 0.001$ by two-sided *t* test.

**(F).** Schematic illustration of NAT10 functional domains and point mutations.

Data represent mean ± SEM from $n = 3$ biological replicates shown as points. Source data are provided in a Source data file.

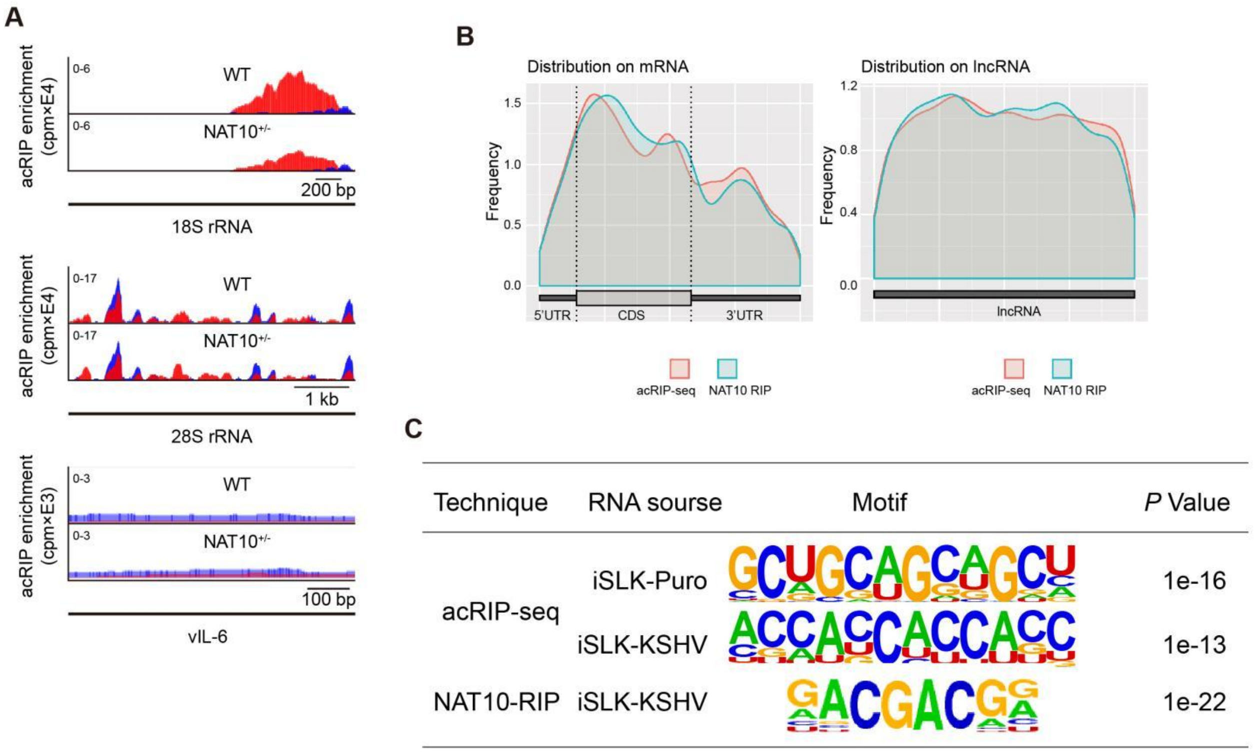


**Supplementary Figure 2. Mappings and motifs of ac$^4$C peaks and NAT10-binding sites.**

**(A).** ac$^4$C peaks in 18S rRNA, 28S rRNA and KSHV vIL-6 transcript were compared between iSLK-KSHV cells with (**NAT10$^{+/-}$**) or without (**WT**) NAT10 knockdown by acRIP-seq. Input and acRIP reads are marked as blue and red, respectively.

**(B).** Metagene analysis of the distribution of acRIP-seq-mapped ac$^4$C sites and RIP-seq-mapped NAT10-bound RNA sites in induced iSLK-KSHV cells, across the untranslated regions (UTRs) and coding regions (CDS) of cellular transcripts (left lane) or lncRNAs (right lane).

**(C).** Enriched sequence motifs in ac$^4$C peaks (**acRIP-seq**, upper lane) and NAT10-binding sites (**NAT10-RIP**, lower lane).

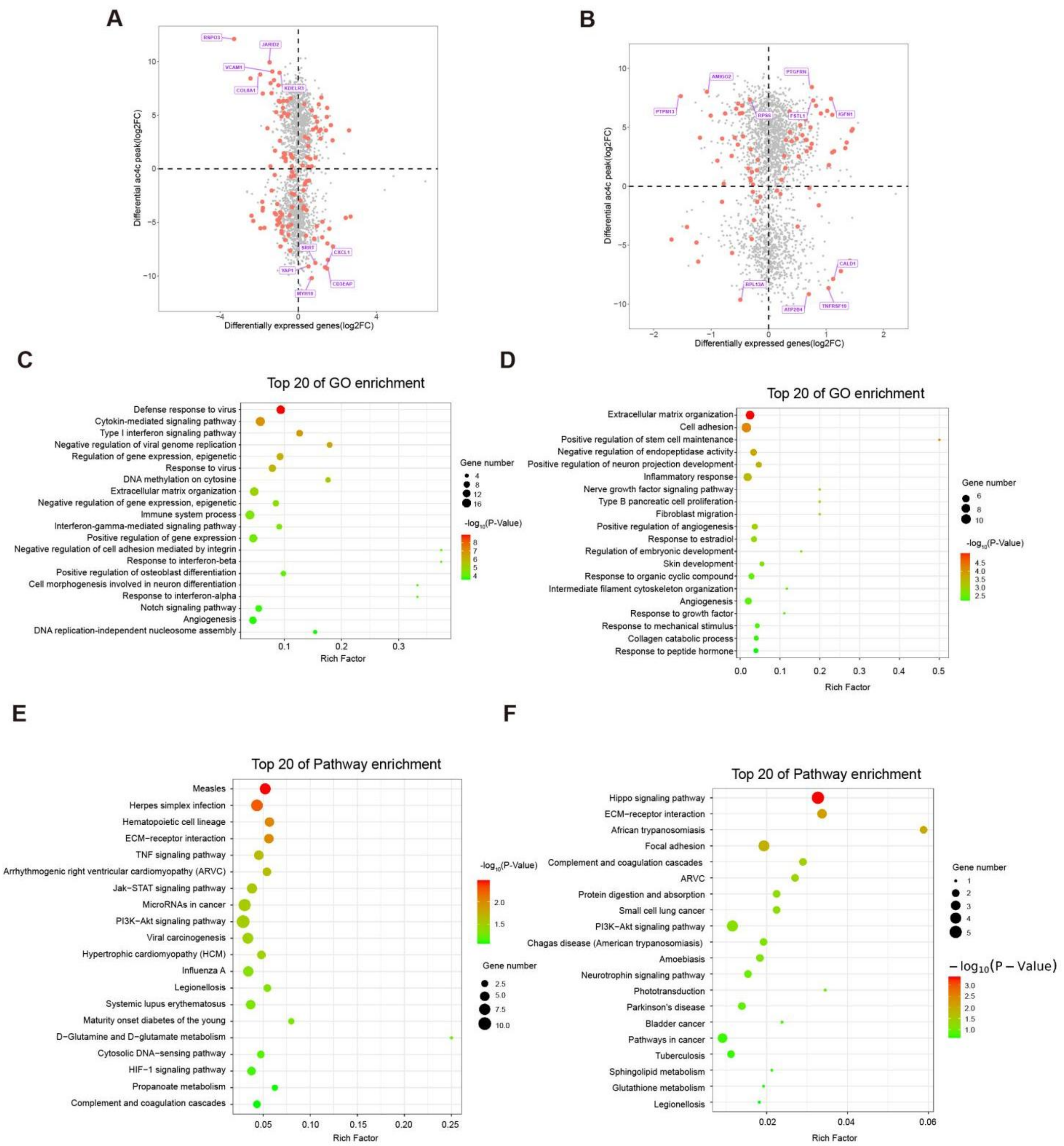
A
B
Differential ac4c peak(log2FC)
Differentially expressed genes(log2FC)
C
Top 20 of GO enrichment
Defense response to virus
Cytokin-mediated signaling pathway
Type I interferon signaling pathway
Negative regulation of viral genome replication
Regulation of gene expression, epigenetic
Response to virus
DNA methylation on cytosine
Extracellular matrix organization
Negative regulation of gene expression, epigenetic
Immune system process
Interferon-gamma-mediated signaling pathway
Positive regulation of gene expression
Negative regulation of cell adhesion mediated by integrin
Response to interferon-beta
Positive regulation of osteoblast differentiation
Cell morphogenesis involved in neuron differentiation
Response to interferon-alpha
Notch signaling pathway
Angiogenesis
DNA replication-independent nucleosome assembly
Rich Factor
Gene number
-log10(P-Value)
D
Top 20 of GO enrichment
Extracellular matrix organization
Cell adhesion
Positive regulation of stem cell maintenance
Negative regulation of endopeptidase activity
Positive regulation of neuron projection development
Inflammatory response
Nerve growth factor signaling pathway
Type B pancreatic cell proliferation
Fibroblast migration
Positive regulation of angiogenesis
Response to estradiol
Regulation of embryonic development
Skin development
Response to organic cyclic compound
Intermediate filament cytoskeleton organization
Angiogenesis
Response to growth factor
Response to mechanical stimulus
Collagen catabolic process
Response to peptide hormone
Rich Factor
Gene number
-log10(P-Value)
E
Top 20 of Pathway enrichment
Measles
Herpes simplex infection
Hematopoietic cell lineage
ECM−receptor interaction
TNF signaling pathway
Arrhythmogenic right ventricular cardiomyopathy (ARVC)
Jak−STAT signaling pathway
MicroRNAs in cancer
PI3K−Akt signaling pathway
Viral carcinogenesis
Hypertrophic cardiomyopathy (HCM)
Influenza A
Legionellosis
Systemic lupus erythematosus
Maturity onset diabetes of the young
D−Glutamine and D−glutamate metabolism
Cytosolic DNA−sensing pathway
HIF−1 signaling pathway
Propanoate metabolism
Complement and coagulation cascades
Rich Factor
-log10(P-Value)
Gene number
F
Top 20 of Pathway enrichment
Hippo signaling pathway
ECM−receptor interaction
African trypanosomiasis
Focal adhesion
Complement and coagulation cascades
ARVC
Protein digestion and absorption
Small cell lung cancer
PI3K−Akt signaling pathway
Chagas disease (American trypanosomiasis)
Amoebiasis
Neurotrophin signaling pathway
Phototransduction
Parkinson's disease
Bladder cancer
Pathways in cancer
Tuberculosis
Sphingolipid metabolism
Glutathione metabolism
Legionellosis
Rich Factor
Gene number
− log10(P − Value)

**Supplementary Figure 3. GO and KEGG enrichment analysis of cellular ac⁴C mRNA regulated by KSHV and NAT10.**

**(A-B).** Volcano plots of top 10 differential mRNAs in iSLK-Puro and iSLK-KSHV cells (**A**), or in NAT10$^{+/+}$ and NAT10$^{+/-}$ iSLK-KSHV cells (**B**).

**(C-D).** Top 20 GO enrichment of dysregulated mRNAs in iSLK-Puro and iSLK-KSHV cells (**C**), or in NAT10$^{+/+}$ and NAT10$^{+/-}$ iSLK-KSHV cells (**D**).

**(E-F).** Top 20 KEGG pathways enrichment of dysregulated mRNAs in iSLK-Puro and iSLK-KSHV cells (**E**), or in NAT10$^{+/+}$ and NAT10$^{+/-}$ iSLK-KSHV cells (**F**).

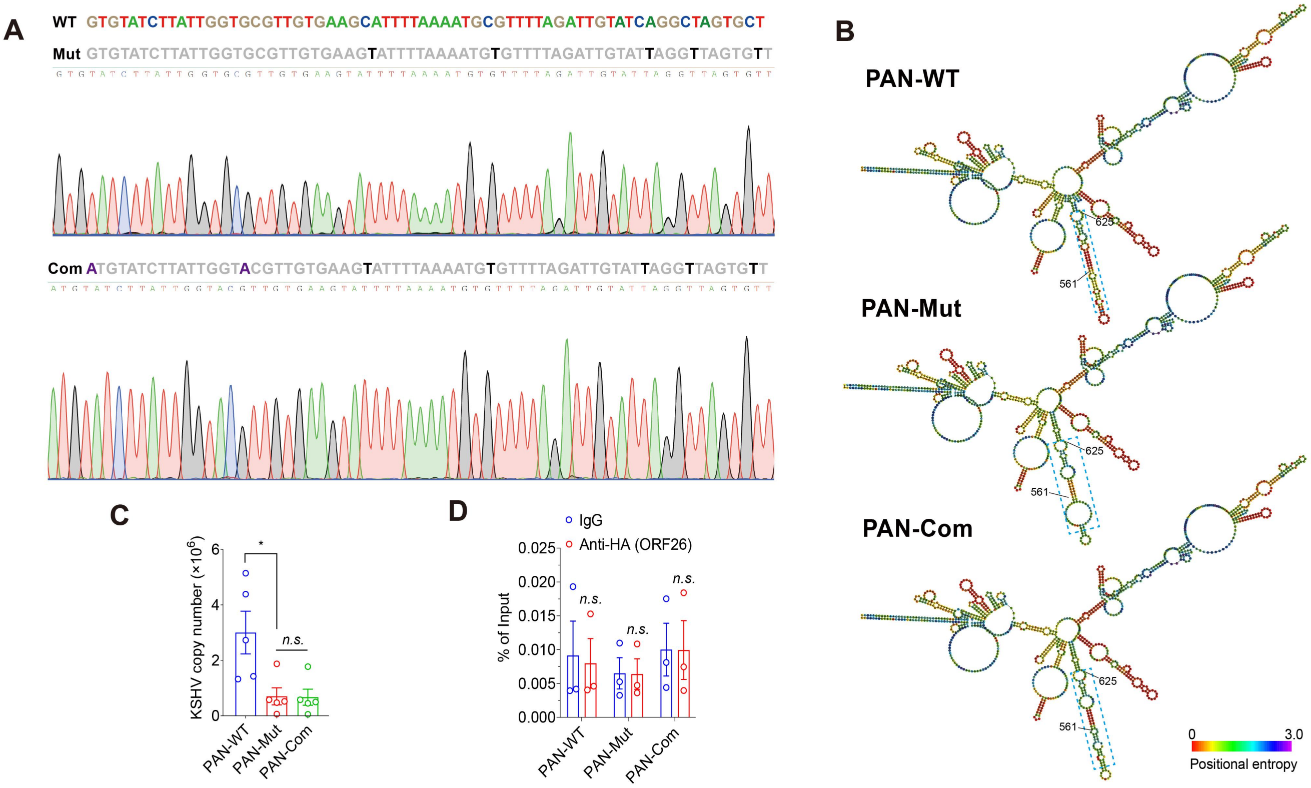
A
WT GTGTATCTTATTGGTGCGTTGTGAAGCATTTTAAAATGCGTTTTAGATTGTATCAGGCTAGTGCT
Mut GTGTATCTTATTGGTGCGTTGTGAAGTATTTTAAAATGTGTTTTAGATTGTATTAGGTTAGTGTT
Com ATGTATCTTATTGGTACGTTGTGAAGTATTTTAAAATGTGTTTTAGATTGTATTAGGTTAGTGTT
B
PAN-WT
PAN-Mut
PAN-Com
625
561
0
3.0
Positional entropy
C
KSHV copy number (×10^6)
*
n.s.
PAN-WT
PAN-Mut
PAN-Com
D
IgG
Anti-HA (ORF26)
% of Input
0.025
0.020
0.015
0.010
0.005
0.000
n.s.

**Supplementary Figure 4. The structure prediction of PAN RNA with mutation.**

**(A).** The $ac^4C$ sites in wild type (**WT**) PAN RNA (561 to 625 nt) were mutated from C (marked as blue) to T (marked as black) to obtain $ac^4C$ mutant PAN (**Mut**), while G561 and G576 in $ac^4C$ mutant PAN were mutated to A576 and A561 (marked as orange) to generate compensated mutant PAN (**Com**). The mutation sites were verified by Sanger sequencing (below).

**(B).** WT PAN (**PAN-WT**), $ac^4C$ mutant PAN (**PAN-Mut**) and compensated mutant PAN (**PAN-Com**) were submitted to the RNAfold web server (http://rna.tbi.univie.ac.at/cgi-bin/RNAWebSuite/RNAfold.cgi) for structure analysis. The blue dashed box indicates the mutation region.

**(C).** iSLK-KSHV cells with WT PAN (**PAN-WT**), $ac^4C$ mutant PAN (**PAN-Mut**) and compensated mutant PAN (**PAN-Com**) were induced by doxycycline for 72 h. Real-time DNA-PCR was then performed to detect viral copy number by the assessment of ORF26. *, $P < 0.05$ by one-way ANOVA. *n.s.*, not significant.

**(D).** The iSLK-Puro cells transduced by HA-tagged KSHV ORF26 were subjected to the anti-HA (ORF26) or immunoglobulin G (IgG) RNA immunoprecipitation, and the WT PAN (**PAN-WT**), $ac^4C$ mutant PAN (**PAN-Mut**) and compensated mutant PAN (**PAN-Com**) were examined by RT-qPCR. *n.s.*, not significant by two-sided *t* test versus the IgG group.

Data represent mean $\pm$ SEM from $n = 3$ (**D**) or $n = 5$ (**C**) biological replicates shown as points. Source data are provided in a Source data file.

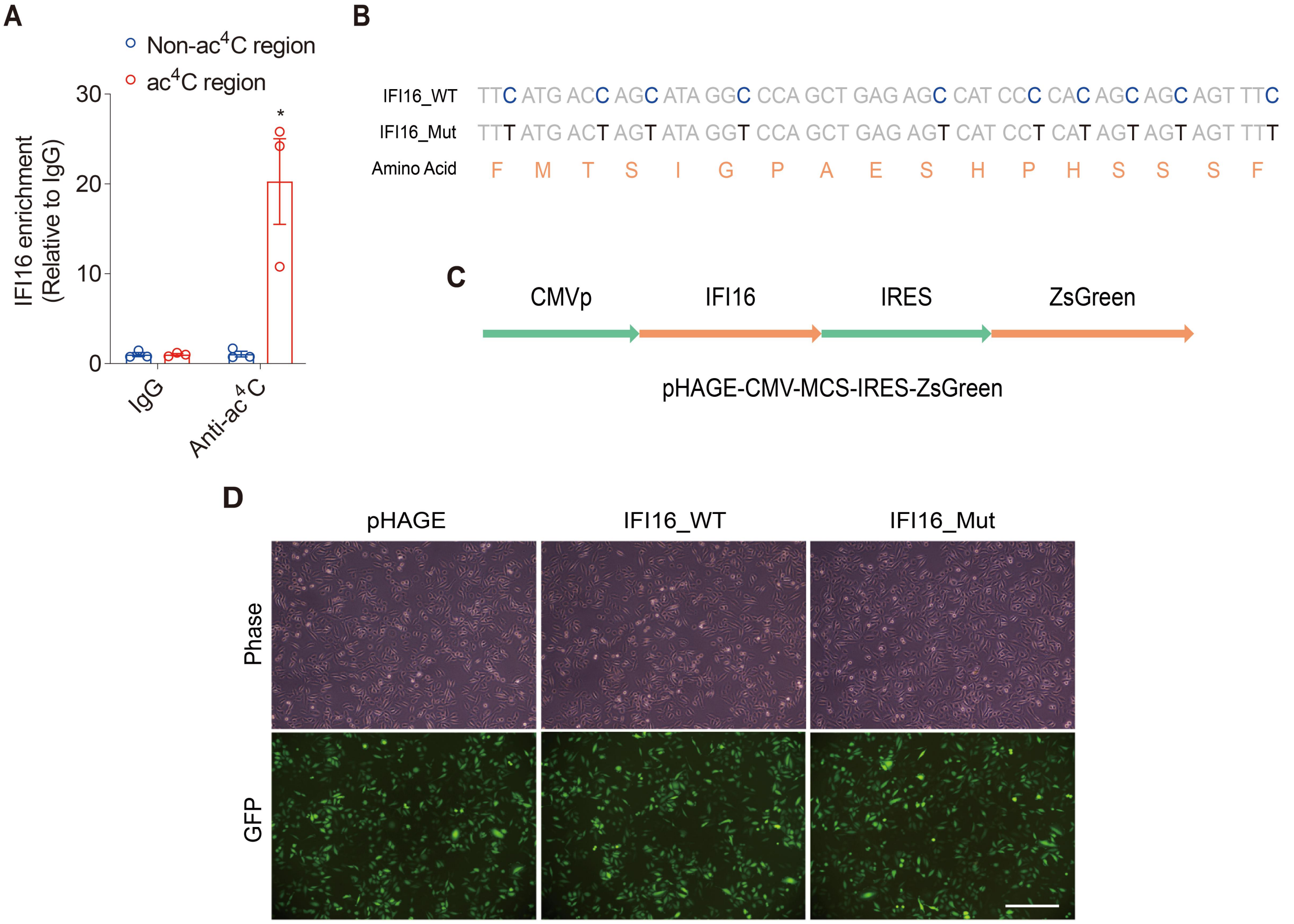
A
Non-ac4C region
ac4C region
IFI16 enrichment (Relative to IgG)
IgG
Anti-ac4C
B
IFI16_WT TTC ATG ACC AGC ATA GGC CCA GCT GAG AGC CAT CCC CAC AGC AGC AGT TTC
IFI16_Mut TTT ATG ACT AGT ATA GGT CCA GCT GAG AGT CAT CCT CAT AGT AGT AGT TTT
Amino Acid F M T S I G P A E S H P H S S S F
C
CMVp
IFI16
IRES
ZsGreen
pHAGE-CMV-MCS-IRES-ZsGreen
D
pHAGE
IFI16_WT
IFI16_Mut
Phase
GFP

**Supplementary Figure 5. Construction of IFI16 mutant into pHAGE plasmid.**

**(A).** RNA immunoprecipitation was performed with anti-$ac^4C$ antibody (**Anti-$ac^4C$**) or IgG (**IgG**), and subsequent RT-qPCR was used to detect $ac^4C$ enrichment on acetylated (**$ac^4C$ region**) or non-acetylated (**Non-$ac^4C$ region**) regions of IFI16 mRNA. *, $P < 0.05$ by two-sided $t$ test versus the non-$ac^4C$ region.

**(B).** $ac^4C$ sites in IFI16 mRNA (exon 9) were mutated from C to T without changing its amino acids sequence in an internal ribozyme entry site (IRES).

**(C).** IFI16 coding sequence was constructed into an IRES based plasmid.

**(D).** The fluorescent intensity was showed after IFI16_WT (**IFI16_WT**) and IFI16_Mut (**IFI16_Mut**) or their control (**pHAGE**) plasmids transfection in HEK293T cells. Scar bars, 40 μm.

Data represent mean ± SEM from $n = 3$ (**A**) biological replicates shown as points. Source data are provided in a Source data file.

**Supplementary Table 1.** Oligonucleotides used for RT-qPCR, PCR and CRISPR/Cas9

| | |
|---|---|
| **RT-qPCR primers** | |
| TGGCGAGGTCAAGCTTAACTTC | KSHV ORF57-F |
| CCCCTGGCCTGTAGTATTCCA | KSHV ORF57-R |
| ATATGTCGCAGGCCGAATAC | KSHV ORF65-F |
| CCACCCATCCTCCTCAGATA | KSHV ORF65-R |
| CGGATTGAGTGTAAATCGGGC | KSHV PAN-F (Also for ac4C-RIP) |
| TGCTTCACAACGCACCAATAAG | KSHV PAN-R (Also for ac4C-RIP) |
| CCGAGGACGAAATGGAAGTG | KSHV LANA (ORF73)-F |
| GGTGATGTTCTGAGTACATAGCGG | KSHV LANA (ORF73)-R |
| AAAGCGTCCAGGCCACCACAGA | KSHV K8.1-F |
| GGCAGAAAATGGCACACGGTTAC | KSHV K8.1-R |
| AGCCGAAAGGATTCCACCAT | ORF26-F |
| GCTGCGGCACGACCAT | ORF26-R |
| TCCAGCAGTTTCTTCACCA | IFI16-F |
| GTCCTGGAAAATGACTCCC | IFI16-R |
| GTATTCTAGAGCCCGCTGCTA | vIL-6-F |
| TTAAATCCTATTAACCCGCAG | vIL-6-R |
| GTCTCTGCGCCATTCAAAAC | vIRF1-F |
| CCGGACACGACAACTAAGAA | vIRF1-R |
| CAACAACCTGCATGGATTCGAG | POLR2H-1F |
| AAGGCTAGCTTCTTCATCAGGA | POLR2H-1R |
| TTTTCCCACATTGGCCTGAGAGC | PAN-F (non-acetylated region) |
| TGAATCCAATGCAATAACCCGCAAG | PAN-R (non-acetylated region) |
| ACAAATAAGCATTGATTCCTGCAT | IFI16-F (non-acetylated region) |
| ATCTTTACAGACATAAGTGAGCCT | IFI16-R (non-acetylated region) |
| | |
| **PCR primers** | |
| TTGCATGACCAGCCCTTTCTAACGCCCCCTTC | Validation of CRISPR editing-F |
| TAGGCATACTCATGGACCCTG | Validation of CRISPR editing-R |
| CGCTTCACCTATGGATTTTGTGCTC | Validation of PAN mutant-F |
| AGCTCTAGGCACGTTAAATTGTCA | Validation of PAN mutant-R |

| | |
|---|---|
| GCCACTTTGCCCTAAATGTGACAATCTGGATGTGTATCTTATTGGTGCGTTGTGAAGTATTTTAAAATGTGTTTTAGATTGTATTAGGTTAGTGTTAGGATGACGACGATAAGTAGGG | Amplification of PAN ac$^4$C mutant-F |
| CTTACACTGGAAAAATAAACACACCATTACAACACTAACCTAATACAATCTAAAACACATTTTAAAATACTTCACAACGCACCAATAAGATACACACAACCAATTAACCAATTCTGATTAG | Amplification of PAN ac$^4$C mutant-R |
| GCCACTTTGCCCTAAATGTGACAATCTGGATATGTATCTTATTGGTACGTTGTGAAGTATTTTAAAATGTGTTTTAGATTGTATTAGGTTAGTGTTAGGATGACGACGATAAGTAGGG | Amplification of PAN compensatory mutant-F |
| CTTACACTGGAAAAATAAACACACCATTACAACACTAACCTAATACAATCTAAAACACATTTTAAAATACTTCACAACGTACCAATAAGATACATACAACCAATTAACCAATTCTGATTAG | Amplification of PAN compensatory mutant-R |
| | |
| **CRISPR guide RNAs for cloning into LentiCRISPRv2** | |
| CCTACGGACCATGAACTCAC | NAT10 |

F, Forward; R, Reverse
