# Supplementary material for: NAT10-dependent N4‐acetylcytidine modification mediates PAN RNA stability, KSHV reactivation, and IFI16-related inflammasome activation
Source: Nat Commun. 2023 Oct 10;14:6327. doi: 10.1038/s41467-023-42135-3 (PMC10564894; doi:10.1038/s41467-023-42135-3)
Supplement: Supplementary file 2 — Reporting Summary [file 41467_2023_42135_MOESM2_ESM.pdf]

nature portfolio

Corresponding author(s): Chun Lu & Xuemei Jia

Last updated by author(s): Sep 3, 2023

Reporting Summary

Nature Portfolio wishes to improve the reproducibility of the work that we publish. This form provides structure for consistency and transparency in reporting. For further information on Nature Portfolio policies, see our Editorial Policies and the Editorial Policy Checklist.

Statistics

For all statistical analyses, confirm that the following items are present in the figure legend, table legend, main text, or Methods section.

n/a	Confirmed	
☐	☒	The exact sample size (*n*) for each experimental group/condition, given as a discrete number and unit of measurement
☐	☒	A statement on whether measurements were taken from distinct samples or whether the same sample was measured repeatedly
☐	☒	The statistical test(s) used AND whether they are one- or two-sided<br>*Only common tests should be described solely by name; describe more complex techniques in the Methods section.*
☒	☐	A description of all covariates tested
☐	☒	A description of any assumptions or corrections, such as tests of normality and adjustment for multiple comparisons
☐	☒	A full description of the statistical parameters including central tendency (e.g. means) or other basic estimates (e.g. regression coefficient) AND variation (e.g. standard deviation) or associated estimates of uncertainty (e.g. confidence intervals)
☐	☒	For null hypothesis testing, the test statistic (e.g. *F*, *t*, *r*) with confidence intervals, effect sizes, degrees of freedom and *P* value noted<br>*Give P values as exact values whenever suitable.*
☒	☐	For Bayesian analysis, information on the choice of priors and Markov chain Monte Carlo settings
☒	☐	For hierarchical and complex designs, identification of the appropriate level for tests and full reporting of outcomes
☒	☐	Estimates of effect sizes (e.g. Cohen's *d*, Pearson's *r*), indicating how they were calculated

Our web collection on statistics for biologists contains articles on many of the points above.

Software and code

Policy information about availability of computer code

Data collection

acRIP-seq data were collected using the Illumina NovaSeq 6000 sequencer. The library products were subjected to NAT10 RIP-seq using Novaseq 6000 sequencer (Illumina) with PE150 model. Images for fluorescent protein were acquired using the Olympus BX53 microscope. FACS data were acquired using BD FACSCalibur with the FlowJo V10.8.0 (BD) and ModFit LT 5.0 (Verity Software House) for cell apoptosis and cell cycle assays, respectively.

Data analysis

For acRIP-seq, the data were processed with Cutadapt (v2.5) to trim adapters and filtered for sequences. Remaining reads were aligned to the human Ensemble genome (GRCh38) by Hisat2 aligner (v2.1.0) with default parameters, which were removed for subsequent alignment to KSHV genome with the modified maximum mismatch penalties (=2). ac4C peaks were identified using exomePeak R package (v2.13.2) under parameters: PEAK_CUTOFF_PVALUE=0.05, PEAK_CUTOFF_FDR=NA, FRAGMENT_LENGTH=200, while the differential ac4C peak were indicated with parameters: PEAK_CUTOFF_PVALUE=0.05, PEAK_CUTOFF_FDR=NA, FRAGMENT_LENGTH=200. ac4C-RNA-related genomic features were visualized using Guitar R package (v1.16.0). ac4C peaks that with P value < 0.05 were chosen for the de novo motif analysis using homer (v4.10.4) under parameters: -len 12 -rna. Raw sequencing data was initially filtered by Trimmomatic (version 0.36).

For protein binding site analysis, the de-duplicated consensus sequences were used and mapped to the reference genome of KSHV (https://www.ncbi.nlm.nih.gov/nuccore/NC_009333.1) using STAR software (version 2.5.3a) with default parameters. RSeQC (version 2.6) and exomePeak (version 3.8) were used for reads distribution analysis and peak calling, respectively. Peaks were annotated by bedtools (version 2.25.0), while the deepTools (version 2.4.1) was used for peak distribution analysis. Using fisher test, the differential binding peaks were identified by a python script. Sequence motifs enriched in peak regions were identified using Homer (version 4.10).

For Gene ontology (GO) analysis and Kyoto encyclopedia of genes and genomes (KEGG) analysis, clusterprofile R package (v3.6.0) was used, and annotated genes were implemented by KOBAS software (version 2.1.1) with a corrected P-value cutoff of 0.05 to judge statistically

significant enrichment.

FACS data were analysed using FlowJo V10.8.0 (BD) and ModFit LT 5.0 (Verity Software House) for cell apoptosis and cell cycle assays, respectively. Statistical data analyses throughout the manuscript were conducted using Graphpad Prism (version 8.0).

For manuscripts utilizing custom algorithms or software that are central to the research but not yet described in published literature, software must be made available to editors and reviewers. We strongly encourage code deposition in a community repository (e.g. GitHub). See the Nature Portfolio guidelines for submitting code & software for further information.

Data

Policy information about availability of data

All manuscripts must include a data availability statement. This statement should provide the following information, where applicable:

- Accession codes, unique identifiers, or web links for publicly available datasets
- A description of any restrictions on data availability
- For clinical datasets or third party data, please ensure that the statement adheres to our policy

The acRIP-seq and NAT10 RIP-seq data generated in this study have been deposited in the NCBI Gene Expression Omnibus (GEO) database under accession number GSE174161 and GSE173889, respectively. The acRIP-seq and NAT10 RIP-seq data based on KSHV genome reference (https://www.ncbi.nlm.nih.gov/datasets/genome/GCF_000838265.1/) and human genome reference (https://www.ncbi.nlm.nih.gov/datasets/genome/GCF_000001405.13/), respectively. All the other data associated with this study are shown in the manuscript, Supplementary Information, and Source Data file. Source data are provided with this paper.

Human research participants

Policy information about studies involving human research participants and Sex and Gender in Research.

Reporting on sex and gender	N/A
Population characteristics	N/A
Recruitment	N/A
Ethics oversight	N/A

Note that full information on the approval of the study protocol must also be provided in the manuscript.

Field-specific reporting

Please select the one below that is the best fit for your research. If you are not sure, read the appropriate sections before making your selection.

[x] Life sciences [ ] Behavioural & social sciences [ ] Ecological, evolutionary & environmental sciences

For a reference copy of the document with all sections, see nature.com/documents/nr-reporting-summary-flat.pdf

Life sciences study design

All studies must disclose on these points even when the disclosure is negative.

Sample size	Sample size for each experiment is indicated in the Figures and Figure legends. No statistical methods were used to predetermine sample size.
Data exclusions	No data have been excluded from the analyses presented in this study.
Replication	All the experiments were repeated at least 3 biological times, and each biological experiment contained 3 or 4 technical replicates, showing the similar results. Data shown represent mean ± SEM from three independent experiments at least. No reproducibility problems found.
Randomization	No randomization procedures were applied in this study. The experiments performed in different mammalian cells with defined NAT10 knockdown, PAN mutations, or lentiviral integrations. A selection bias does not affect the results in these studies. Samples and their appropriate controls are processed at the same time.
Blinding	For microscopy images, the investigators were blinded to group allocation during data collection and analysis. Other studies were not blinded because they did not involve subjective measurements. The relevant controls were processed at the same time as the samples and read in an unbiased manner.

Behavioural & social sciences study design

All studies must disclose on these points even when the disclosure is negative.

Study description	*Briefly describe the study type including whether data are quantitative, qualitative, or mixed-methods (e.g. qualitative cross-sectional, quantitative experimental, mixed-methods case study).*
Research sample	*State the research sample (e.g. Harvard university undergraduates, villagers in rural India) and provide relevant demographic information (e.g. age, sex) and indicate whether the sample is representative. Provide a rationale for the study sample chosen. For studies involving existing datasets, please describe the dataset and source.*
Sampling strategy	*Describe the sampling procedure (e.g. random, snowball, stratified, convenience). Describe the statistical methods that were used to predetermine sample size OR if no sample-size calculation was performed, describe how sample sizes were chosen and provide a rationale for why these sample sizes are sufficient. For qualitative data, please indicate whether data saturation was considered, and what criteria were used to decide that no further sampling was needed.*
Data collection	*Provide details about the data collection procedure, including the instruments or devices used to record the data (e.g. pen and paper, computer, eye tracker, video or audio equipment) whether anyone was present besides the participant(s) and the researcher, and whether the researcher was blind to experimental condition and/or the study hypothesis during data collection.*
Timing	*Indicate the start and stop dates of data collection. If there is a gap between collection periods, state the dates for each sample cohort.*
Data exclusions	*If no data were excluded from the analyses, state so OR if data were excluded, provide the exact number of exclusions and the rationale behind them, indicating whether exclusion criteria were pre-established.*
Non-participation	*State how many participants dropped out/declined participation and the reason(s) given OR provide response rate OR state that no participants dropped out/declined participation.*
Randomization	*If participants were not allocated into experimental groups, state so OR describe how participants were allocated to groups, and if allocation was not random, describe how covariates were controlled.*

Ecological, evolutionary & environmental sciences study design

All studies must disclose on these points even when the disclosure is negative.

Study description	*Briefly describe the study. For quantitative data include treatment factors and interactions, design structure (e.g. factorial, nested, hierarchical), nature and number of experimental units and replicates.*
Research sample	*Describe the research sample (e.g. a group of tagged Passer domesticus, all Stenocereus thurberi within Organ Pipe Cactus National Monument), and provide a rationale for the sample choice. When relevant, describe the organism taxa, source, sex, age range and any manipulations. State what population the sample is meant to represent when applicable. For studies involving existing datasets, describe the data and its source.*
Sampling strategy	*Note the sampling procedure. Describe the statistical methods that were used to predetermine sample size OR if no sample-size calculation was performed, describe how sample sizes were chosen and provide a rationale for why these sample sizes are sufficient.*
Data collection	*Describe the data collection procedure, including who recorded the data and how.*
Timing and spatial scale	*Indicate the start and stop dates of data collection, noting the frequency and periodicity of sampling and providing a rationale for these choices. If there is a gap between collection periods, state the dates for each sample cohort. Specify the spatial scale from which the data are taken*
Data exclusions	*If no data were excluded from the analyses, state so OR if data were excluded, describe the exclusions and the rationale behind them, indicating whether exclusion criteria were pre-established.*
Reproducibility	*Describe the measures taken to verify the reproducibility of experimental findings. For each experiment, note whether any attempts to repeat the experiment failed OR state that all attempts to repeat the experiment were successful.*
Randomization	*Describe how samples/organisms/participants were allocated into groups. If allocation was not random, describe how covariates were controlled. If this is not relevant to your study, explain why.*
Blinding	*Describe the extent of blinding used during data acquisition and analysis. If blinding was not possible, describe why OR explain why blinding was not relevant to your study.*

Did the study involve field work? ☐ Yes ☐ No

Field work, collection and transport

Field conditions	*Describe the study conditions for field work, providing relevant parameters (e.g. temperature, rainfall).*
Location	*State the location of the sampling or experiment, providing relevant parameters (e.g. latitude and longitude, elevation, water depth).*
Access & import/export	*Describe the efforts you have made to access habitats and to collect and import/export your samples in a responsible manner and in compliance with local, national and international laws, noting any permits that were obtained (give the name of the issuing authority, the date of issue, and any identifying information).*
Disturbance	*Describe any disturbance caused by the study and how it was minimized.*

Reporting for specific materials, systems and methods

We require information from authors about some types of materials, experimental systems and methods used in many studies. Here, indicate whether each material, system or method listed is relevant to your study. If you are not sure if a list item applies to your research, read the appropriate section before selecting a response.

Materials & experimental systems

n/a	Involved in the study	
☐	☒	Antibodies
☐	☒	Eukaryotic cell lines
☒	☐	Palaeontology and archaeology
☒	☐	Animals and other organisms
☒	☐	Clinical data
☒	☐	Dual use research of concern

Methods

n/a	Involved in the study	
☒	☐	ChIP-seq
☐	☒	Flow cytometry
☒	☐	MRI-based neuroimaging

Antibodies

Antibodies used

Anti-GAPDH (sc-47724; 1: 500), anti-beta-actin (sc-47778; 1: 500) and anti-Tubulin (sc-23948; 1: 500) were purchased from Santa Cruz Biotechnology. Anti-Flag was purchased from MBL (M185-3L, Tokyo, Japan; 1: 1 000). Antibodies against NAT10 (ab194297; 1: 1 000), LANA (ab4103; 1: 1 000), IFI16 (ab169788; 1: 1 000) and ac4C (ab252215; 1: 1000) were from Abcam. Anti-pro Caspase-1+p10 +p12 (ab179515; 1: 1 000) was also obtained from Abcam. IL-1β (3A6) mouse mAb (12242; 1: 1 000) and cleaved Caspase-1 (Asp296) (E2G2I) rabbit mAb (89332; 1: 1 000) were from Cell Signaling Technology. Anti-vIL-6 (1: 500) and anti-vIRF1 (1: 300) antibodies were provided by Dr. Robert Yarchoan (National Cancer Institute) and Dr. Gary Hayward (Johns Hopkins School of Medicine), respectively.

Validation

Anti-GAPDH (sc-47724, Santa Cruz Biotechnology): https://www.scbt.com/p/gapdh-antibody-0411

Anti-beta-actin (sc-47778, Santa Cruz Biotechnology):
https://www.scbt.com/p/beta-actin-antibody-c4?gclid=EAIaIQobChMIrLKY58SGgQMVXsZMAh2EhAecEAAYAiAAEgLZyvD_BwE

Anti-Tubulin (sc-23948, Santa Cruz Biotechnology):
https://www.scbt.com/p/alpha-tubulin-antibody-b-5-1-2

Anti-Flag (M185-3L, MBL):
https://www.mblbio.com/bio/g/dtl/A/?pcd=M185-3L

Anti-NAT10 (ab194297, Abcam):
https://www.abcam.com/products/primary-antibodies/nat10-antibody-epr18663-ab194297.html

Anti-LANA (ab4103, Abcam):
https://www.abcam.com/products/primary-antibodies/hhv8-antibody-ln53-ab4103.html

Anti-IFI16 (ab169788, Abcam):
https://www.abcam.com/products/primary-antibodies/ifi16-antibody-epr11767b-ab169788.html

Anti-ac4C (ab252215, Abcam):
https://www.abcam.com/products/primary-antibodies/n4-acetylcytidine-ac4c-antibody-eprnci-184-128-ab252215.html

Anti-pro Caspase-1+p10+p12 (ab179515, Abcam):
https://www.abcam.com/products/primary-antibodies/pro-caspase-1--p10--p12-antibody-epr16883-ab179515.html

Anti-IL-1β (3A6) mouse mAb (12242, Cell Signaling Technology):
https://www.cellsignal.com/products/primary-antibodies/il-1b-3a6-mouse-mab/12242?_requestid=652733

Anti-cleaved Caspase-1 (Asp296) (E2G2I) rabbit mAb (89332, Cell Signaling Technology):
https://www.cellsignal.com/products/primary-antibodies/cleaved-caspase-1-asp296-e2g2i-rabbit-mab/89332

Anti-vIL-6 was provided by Dr. Robert Yarchoan (Duosha Hu et al. J Virol 2015).

Anti-vIRF1 was provided by Dr. Gary Hayward (Wan Li et al. Cell Death Differ 2020).

Eukaryotic cell lines

Policy information about cell lines and Sex and Gender in Research

Cell line source(s)	iSLK-KSHV cells and iSLK-Puro cells were kindly provided by Prof. Ke Lan (Wuhan University).<br>HEK293T cells were purchased from ATCC (CRL-11268).
Authentication	iSLK-Puro cells were selected with puromycin and G418, while iSLK-KSHV cells were selected with puromycin, G418 and hygromycin B. Titers of KSHV stocks were determined by infecting serially diluted stocks on HEK293T cells with subsequent enumeration of GFP-expressing cells by FACS analysis. KSHV genome in iSLK-KSHV cells were sequenced via PCR amplification based on ORF26, ORF11 and ORF69.<br>HEK293T cells were commercially purchased by ATCC. The manufacturer tested and authenticated HEK293T cells.
Mycoplasma contamination	All cell lines were routinely tested negative for mycoplasma contamination.
Commonly misidentified lines<br>(See ICLAC register)	No commonly misidentified cell lines were used in this study.

Palaeontology and Archaeology

Specimen provenance	*Provide provenance information for specimens and describe permits that were obtained for the work (including the name of the issuing authority, the date of issue, and any identifying information). Permits should encompass collection and, where applicable, export.*
Specimen deposition	*Indicate where the specimens have been deposited to permit free access by other researchers.*
Dating methods	*If new dates are provided, describe how they were obtained (e.g. collection, storage, sample pretreatment and measurement), where they were obtained (i.e. lab name), the calibration program and the protocol for quality assurance OR state that no new dates are provided.*

☐ Tick this box to confirm that the raw and calibrated dates are available in the paper or in Supplementary Information.

Ethics oversight	*Identify the organization(s) that approved or provided guidance on the study protocol, OR state that no ethical approval or guidance was required and explain why not.*

Note that full information on the approval of the study protocol must also be provided in the manuscript.

Animals and other research organisms

Policy information about studies involving animals; ARRIVE guidelines recommended for reporting animal research, and Sex and Gender in Research

Laboratory animals	*For laboratory animals, report species, strain and age OR state that the study did not involve laboratory animals.*
Wild animals	*Provide details on animals observed in or captured in the field; report species and age where possible. Describe how animals were caught and transported and what happened to captive animals after the study (if killed, explain why and describe method; if released, say where and when) OR state that the study did not involve wild animals.*
Reporting on sex	*Indicate if findings apply to only one sex; describe whether sex was considered in study design, methods used for assigning sex. Provide data disaggregated for sex where this information has been collected in the source data as appropriate; provide overall numbers in this Reporting Summary. Please state if this information has not been collected. Report sex-based analyses where performed, justify reasons for lack of sex-based analysis.*
Field-collected samples	*For laboratory work with field-collected samples, describe all relevant parameters such as housing, maintenance, temperature, photoperiod and end-of-experiment protocol OR state that the study did not involve samples collected from the field.*
Ethics oversight	*Identify the organization(s) that approved or provided guidance on the study protocol, OR state that no ethical approval or guidance was required and explain why not.*

Note that full information on the approval of the study protocol must also be provided in the manuscript.

Clinical data

Policy information about clinical studies

All manuscripts should comply with the ICMJE guidelines for publication of clinical research and a completed CONSORT checklist must be included with all submissions.

Clinical trial registration	*Provide the trial registration number from ClinicalTrials.gov or an equivalent agency.*
Study protocol	*Note where the full trial protocol can be accessed OR if not available, explain why.*
Data collection	*Describe the settings and locales of data collection, noting the time periods of recruitment and data collection.*
Outcomes	*Describe how you pre-defined primary and secondary outcome measures and how you assessed these measures.*

Dual use research of concern

Policy information about dual use research of concern

Hazards

Could the accidental, deliberate or reckless misuse of agents or technologies generated in the work, or the application of information presented in the manuscript, pose a threat to:

No	Yes	
☐	☐	Public health
☐	☐	National security
☐	☐	Crops and/or livestock
☐	☐	Ecosystems
☐	☐	Any other significant area

Experiments of concern

Does the work involve any of these experiments of concern:

No	Yes	
☐	☐	Demonstrate how to render a vaccine ineffective
☐	☐	Confer resistance to therapeutically useful antibiotics or antiviral agents
☐	☐	Enhance the virulence of a pathogen or render a nonpathogen virulent
☐	☐	Increase transmissibility of a pathogen
☐	☐	Alter the host range of a pathogen
☐	☐	Enable evasion of diagnostic/detection modalities
☐	☐	Enable the weaponization of a biological agent or toxin
☐	☐	Any other potentially harmful combination of experiments and agents

ChIP-seq

Data deposition

☐ Confirm that both raw and final processed data have been deposited in a public database such as GEO.

☐ Confirm that you have deposited or provided access to graph files (e.g. BED files) for the called peaks.

Data access links<br>*May remain private before publication.*	*For "Initial submission" or "Revised version" documents, provide reviewer access links. For your "Final submission" document, provide a link to the deposited data.*
Files in database submission	*Provide a list of all files available in the database submission.*
Genome browser session<br>(e.g. UCSC)	*Provide a link to an anonymized genome browser session for "Initial submission" and "Revised version" documents only, to enable peer review. Write "no longer applicable" for "Final submission" documents.*

Methodology

Replicates	*Describe the experimental replicates, specifying number, type and replicate agreement.*
Sequencing depth	*Describe the sequencing depth for each experiment, providing the total number of reads, uniquely mapped reads, length of reads and whether they were paired- or single-end.*

Antibodies	*Describe the antibodies used for the ChIP-seq experiments; as applicable, provide supplier name, catalog number, clone name, and lot number.*
Peak calling parameters	*Specify the command line program and parameters used for read mapping and peak calling, including the ChIP, control and index files used.*
Data quality	*Describe the methods used to ensure data quality in full detail, including how many peaks are at FDR 5% and above 5-fold enrichment.*
Software	*Describe the software used to collect and analyze the ChIP-seq data. For custom code that has been deposited into a community repository, provide accession details.*

Flow Cytometry

Plots

Confirm that:

- [x] The axis labels state the marker and fluorochrome used (e.g. CD4-FITC).
- [x] The axis scales are clearly visible. Include numbers along axes only for bottom left plot of group (a 'group' is an analysis of identical markers).
- [x] All plots are contour plots with outliers or pseudocolor plots.
- [x] A numerical value for number of cells or percentage (with statistics) is provided.

Methodology

Sample preparation	For cell cycle assay, the indicated cells (2 × 106) were inoculated into a 6-well plate and cultured overnight. Next day, the supernatant was discarded and cells were then trypsinized, centrifuged at 800 rpm for 5 min. Then the cell precipitation was washed twice with pre-cooled PBS, added pre-cooled 75% ethanol, and fixed at 4°C for more than 4 h. Next, centrifuge at 1500 rpm for 5 min, wash with pre-cooled PBS, add 400 μL propidium iodide (PI, 50 μg/mL) and 100 μL RNase A (100 μg/mL), and incubate in dark at 4°C for 30 min. Cell cycle were recorded using BD FACSCalibur, and analyzed by ModFit LT 5.0 (Verity Software House).<br>Cell apoptosis quantification was performed by Annexin V-FITC Apoptosis Detection Kit (C1062, Beyotime). The indicated cells were washed with PBS and then digested with trypsin. 195 μL Annexin V-FITC binding buffer and 5 μL FITC-labeled Annexin V were added and incubated for 10 min at room temperature. Then cells were incubated with 10 μL PI for 10 min on ice in the dark and measured by BD FACSCalibur, and analyzed by FlowJo V10.8.0 (BD).
Instrument	BD FACSCalibur, CellQuest Pro
Software	Cell cycle assays: ModFit LT 5.0 (Verity Software House)<br>Cell apoptosis assays: FlowJo V10.8.0 (BD)
Cell population abundance	Data analyses were performed using at least 10,000 cells in each analysis.
Gating strategy	The gating strategy was defined as compared to non-stained cells. The red fluorescence signal was detected at the excitation wavelength of 488 nm to reflect the DNA content by fluorescence, and then judge the time phase of the cells. Unstained blank cells were used to set up parameters such as voltage and so on.

- [x] Tick this box to confirm that a figure exemplifying the gating strategy is provided in the Supplementary Information.

Magnetic resonance imaging

Experimental design

Design type	*Indicate task or resting state; event-related or block design.*
Design specifications	*Specify the number of blocks, trials or experimental units per session and/or subject, and specify the length of each trial or block (if trials are blocked) and interval between trials.*
Behavioral performance measures	*State number and/or type of variables recorded (e.g. correct button press, response time) and what statistics were used to establish that the subjects were performing the task as expected (e.g. mean, range, and/or standard deviation across subjects).*

Acquisition

Imaging type(s) | *Specify: functional, structural, diffusion, perfusion.*

Field strength | *Specify in Tesla*

Sequence & imaging parameters | *Specify the pulse sequence type (gradient echo, spin echo, etc.), imaging type (EPI, spiral, etc.), field of view, matrix size, slice thickness, orientation and TE/TR/flip angle.*

Area of acquisition | *State whether a whole brain scan was used OR define the area of acquisition, describing how the region was determined.*

Diffusion MRI ☐ Used ☐ Not used

Preprocessing

Preprocessing software | *Provide detail on software version and revision number and on specific parameters (model/functions, brain extraction, segmentation, smoothing kernel size, etc.).*

Normalization | *If data were normalized/standardized, describe the approach(es): specify linear or non-linear and define image types used for transformation OR indicate that data were not normalized and explain rationale for lack of normalization.*

Normalization template | *Describe the template used for normalization/transformation, specifying subject space or group standardized space (e.g. original Talairach, MNI305, ICBM152) OR indicate that the data were not normalized.*

Noise and artifact removal | *Describe your procedure(s) for artifact and structured noise removal, specifying motion parameters, tissue signals and physiological signals (heart rate, respiration).*

Volume censoring | *Define your software and/or method and criteria for volume censoring, and state the extent of such censoring.*

Statistical modeling & inference

Model type and settings | *Specify type (mass univariate, multivariate, RSA, predictive, etc.) and describe essential details of the model at the first and second levels (e.g. fixed, random or mixed effects; drift or auto-correlation).*

Effect(s) tested | *Define precise effect in terms of the task or stimulus conditions instead of psychological concepts and indicate whether ANOVA or factorial designs were used.*

Specify type of analysis: ☐ Whole brain ☐ ROI-based ☐ Both

Statistic type for inference
(See Eklund et al. 2016) | *Specify voxel-wise or cluster-wise and report all relevant parameters for cluster-wise methods.*

Correction | *Describe the type of correction and how it is obtained for multiple comparisons (e.g. FWE, FDR, permutation or Monte Carlo).*

Models & analysis

n/a	Involved in the study
☐	☐ Functional and/or effective connectivity
☐	☐ Graph analysis
☐	☐ Multivariate modeling or predictive analysis

Functional and/or effective connectivity | *Report the measures of dependence used and the model details (e.g. Pearson correlation, partial correlation, mutual information).*

Graph analysis | *Report the dependent variable and connectivity measure, specifying weighted graph or binarized graph, subject- or group-level, and the global and/or node summaries used (e.g. clustering coefficient, efficiency, etc.).*

Multivariate modeling and predictive analysis | *Specify independent variables, features extraction and dimension reduction, model, training and evaluation metrics.*
