# Supplementary material for: NAT10-dependent N4‐acetylcytidine modification mediates PAN RNA stability, KSHV reactivation, and IFI16-related inflammasome activation
Source: Nat Commun. 2023 Oct 10;14:6327. doi: 10.1038/s41467-023-42135-3 (PMC10564894; doi:10.1038/s41467-023-42135-3)
Supplement: Supplementary file 3 — Description of Additional Supplementary Files [file 41467_2023_42135_MOESM3_ESM.pdf]

Description of Additional Supplementary Files

Supplementary Data 1: The detailed results for acRIP-seq.
